# A comparative study on recombination activity in cattle

**DOI:** 10.1186/s12711-026-01041-0

**Published:** 2026-04-06

**Authors:** Dörte Wittenburg, Nina Melzer, Nayyer Abdollahi Sisi, Xi Ding, Franz R. Seefried

**Affiliations:** 1 Research Institute for Farm Animal Biology (FBN), 18196 Dummerstorf, Germany; 2 Qualitas AG, 6300 Zug, Switzerland

## Abstract

**Background:**

Genetic diversity among cattle breeds exists due to their demographic history and different breeding objectives for meat, dairy or dual purpose. Taking this into account, targeted breeding strategies can be further enhanced by employing breed-specific genetic maps. The aim of this study was to derive genetic maps (also known as linkage maps) for a selection of commercial breeds.

**Results:**

We analysed genotype data from six cattle breeds with sample size ranging from 4,181 to 76,875. Several different assays were used for genotyping, resulting in a high proportion of systematically missing data. Thus, we streamlined the data preparation and analysed the data with three different approaches. We investigated the frequency of paternal and maternal recombination events and derived genetic-map coordinates of about 50K SNP markers. Estimates of male map length varied from 26.97 to 29.83 M between breeds, whereas length of female maps ranged from 23.32 to 26.08 M. Female recombination activity and female genetic maps were clearly distinct from the outcome in males. In particular, male maps of Brown Swiss, Simmental and Angus clustered separately from other male maps and female maps of dairy breeds were distinct from female maps of dual-purpose breeds. Furthermore, a genomewide association study on mean autosomal crossover count and intra-chromosomal allelic shuffling per parent revealed two chromosomal regions on BTA6 and BTA10 with strong evidence found in Brown Swiss and Swiss Holstein; another suggestive signal was detected on BTA10. These regions were in close proximity to genes with known impact on recombination activity.

**Conclusion:**

Breed-specific female maps could be clearly differentiated from male maps. Though female maps clustered together depending on breed purpose, this was not the case for male maps. All results on empirical data were implemented in the updated version of the R Shiny app CLARITY v3.0.0. This resource can contribute to several instances of genomic evaluations – from improving haplotype phasing to implementing novel breeding strategies.

## Background

The domestication of cattle can be dated back thousands of years and manifold cattle breeds have been generated since then [9]. Being directly associated with its economic importance, the population sizes differ largely, ranging from few hundreds or less in local breeds to hundreds of thousands in worldwide commercially dominating breeds such as Holstein in dairy industry or Black Angus in beef production [14]. As the breeding goals differ, the genetic make-up of these breeds may vary accordingly after decades of targeted selection for dairy, beef or dual-purpose characteristics. Studies on genetic diversity (e.g. [20, 33]) and phylogenetics (e.g.[12]) could clearly differentiate cattle populations and their demographic history. Based on a common reference genome retrieved from Hereford cattle [46], breed-specific DNA sequences and private polymorphisms were found [11]. Consequently, not only the distance between shared polymorphisms in physical units (base pairs, bp) can vary between breeds but probably also the distance in genetic-map units (Morgan, M). The latter is of utmost importance in animal breeding as it is related to the probability that non-/recombinant haplotype blocks are transmitted from parent to progeny. Hence, the genetic distance between loci allows drawing inferences, for instance, on genetic variation of not yet born progeny and provides valuable information when searching for top breeding animals [3] or useful crosses [30, 55]. Following this idea, Musa and Reinsch [37] have used genetic maps for deriving haplotype similarity among hypothetical progeny of mating candidates to identify most valuable matings.

The objective of this study was to derive genetic-map coordinates for different dairy, beef and dual-purpose breeds from a medium density panel of single nucleotide polymorphisms (SNPs). Since data collection strategies differed among breeds in terms of array used or parents genotyped, the genotype data differed in their completeness. We evaluated the quality of genetic maps derived by undertaking additional simulation studies. Furthermore, the number of recombination events (i.e. autosomal crossover count) and the proportion of intra-chromosomal allelic shuffling were considered as parental traits of recombination activity and sex- and breed-specific differences were assessed. Additionally, a genomewide association study (GWAS) was performed for these traits to identify genome regions that are potentially linked with recombination activity. To complement this study, the R Shiny app CLARITY [35] has been essentially revised, providing access to a wider collection of bovine genetic maps.

## Data

This study comprises genotype data of dairy (Brown Swiss and Swiss Holstein; Holstein-CH), beef (Limousin and Angus) and dual-purpose breeds (Original Braunvieh and Simmental) which have been provided by Qualitas AG (Zug, Switzerland). Hereby, the Brown Swiss population also encloses the Braunvieh population that has been derived from Original Braunvieh by introgression of Brown Swiss since decades [51]. In each breed, various SNP arrays with marker density varying from 6,607 to 125,839 SNPs were used for genotyping, inducing a high degree of systematically missing data. A SNP sub-panel corresponding to the design “chip 4” was selected for further investigations. This sub-panel encompasses a group of publicly available genotyping arrays where each array contains between 42K and 58K markers. Core group members providing most group data are Illumina Bovine SNP50 array v1 and v2 (Illumina Inc., San Diego, CA). An overview of total genotyped individuals, genotyped parents, number of half-sib families and number of SNPs used per breed after quality control, which is described in the next section, is given in Table 1. The maximum size of paternal (maternal) half-sib families varied strongly among breeds: 1,803 (50) in Holstein-CH, 2,663 (37) in Brown Swiss, 284 (15) in Simmental, 236 (21) in Original Braunvieh, 95 (9) in Limousin and 68 (21) in Angus. The mean size of paternal half-sib families was: 16 in Holstein-CH, 23 in Brown Swiss, 6 in Simmental, 7 in Original Braunvieh, 5 in Limousin and 4 in Angus. On average 1-2 progeny were born per dam.

**Table 1:** Summary about genotyped individuals per breed

Breed	$n_t$	*N*	$n_s$	$n_d$	$n^*_t$	$N^*$	*q*	Missing
Holstein-CH	76,875	4,891	2,869	17,450	57,462	484	51,603	0.478
Brown Swiss	68,050	2,958	2,426	12,587	55,654	308	49,880	0.468
Simmental	13,406	2,127	1,286	2,181	5,693	68	50,303	0.430
Original Braunvieh	12,604	1,723	1,112	2,643	5,918	79	51,182	0.341
Limousin	6,842	1,396	706	331	1,718	39	44,466	0.151
Angus	4,181	1,067	463	270	506	11	45,047	0.222

$n_t$ the total number of genotyped animals, *N* the total number of paternal half-sib families, $n_s$ the number of genotyped sires, $n_d$ the number of genotyped dams, $n^*_t$ the number of genotyped animals in half-sib families with at least 30 progeny, $N^*$ the number of paternal half-sib families with at least 30 progeny, *q* the number of SNPs for recombination rate analysis, and the proportion of missing genotypes among $n_t$ individuals averaged over chromosomes

## Methods

### Estimation of recombination rate and derivation of genetic map

A standardised workflow similarly to Qanbari and Wittenburg [42] and Melzer et al. [35] was applied to each breed: markers were ordered according to the ARS-UCD1.2 genome assembly [46]. Markers were excluded if they lie in problematic regions or if they match with candidates of misplacement found in German Holstein (Holstein-DE; [43]). Then, data were filtered for minor allele frequency $>0.01$ and checked for Mendelian inconsistencies with PLINK v1.9 [41]. Families with more than 5 % and SNPs with more than 10 % Mendel errors were excluded. Quality control was completed by setting those genotypes to unknown status where Mendelian inconsistency was still present. Afterwards, recombination rate was estimated with the LINKPHASE3 approach [13], which combines Mendelian segregation and linkage information with a hidden Markov model (HMM; HMM-based approach). This approach enables haplotype phasing as well as crossover identification and additionally provides estimates of sex-specific recombination rates according to Zhang et al. [57]. As an example, Additional File 1 (Fig. S1) visualises the occurrence of systematically missing genotypes on BTA1 in Simmental after quality control, revealing the challenge of haplotype phasing to derive recombination rates later on. After an initial run of LINKPHASE3, markers which received an internal map confidence score (MCS) of $\ge 0.986$ and individuals with $\le 58$ crossovers were further considered and a second run of LINKPHASE3 was started to obtain the final estimates. Genetic-map coordinates in Morgan units were obtained as accumulation of recombination rate between adjacent SNPs.

Furthermore, two methods, which yielded highly accurate estimates of male recombination rates in a Holstein-DE population consisting of large paternal half-sib families [42], were used for comparison: a deterministic (R package hsphase v2.0.2; [15, 16]) and a likelihood-based approach (R package hsrecombi 1.0.1, [22, 54]). These two approaches required a reduction of genotype data towards paternal half-sib families with $\ge 30$ genotyped progeny to ensure that sire haplotypes were precisely estimated from progeny genotypes. The number of genotyped individuals remaining ($n^*_t$) is also given in Table 1. The deterministic approach is a multipoint method taking by default 30 heterozygous loci into account, whereas the likelihood-based approach solely works on linkage and linkage disequilibrium between intra-chromosomal pairs of markers. Hence, the HMM-based method, which incorporates likelihood inferences on multiple loci, should deliver similar or better outcome than the other two methods.

To investigate similarities between breed- and sex-specific recombination maps, recombination rates between adjacent SNPs and genetic-map coordinates obtained from the HMM-based approach were inspected. Only SNPs shared between all breeds were included. The principal components of the recombination matrix (one line of recombination rates for each sex and breed) were determined using the R function pca from package dimensio v0.14.0 [17]. Furthermore, hierarchical clustering of sex- and breed-specific maps was performed with use of the R function hclust from package stats v4.4.0 [45], taking into account the L$_2$ norm of differences of genetic-map coordinates of SNPs.

Unless otherwise specified, we used R v4.2.1 [44] on x86_64-suse-linux-gnu for computation.

### Simulation study

Due to the nature of artificial insemination, the number and size of paternal half-sib families are key parameters in cattle breeding. Since these parameters differ substantially among breeds (Table 1), their impact on the accuracy of the estimated male genetic map was explored with use of simulated data. In addition, the influence of the number of markers and of missing data on the estimated male map length was verified. These evaluations help understanding the reliability of results.

*Synthetic population.* A breeding population was simulated with use of the R package AlphaSimR v1.3.2 [18, 19], employing default parameters and with 1,000 individuals constituting the founder population. In total, 4,000 markers were distributed on a single chromosome of 1 M length. Mating was at random between 500 sires and 500 females to produce $500 \times {1,000}$ progeny in total. Then, for estimating recombination events, 96 scenarios were obtained as subsets from the full data: the number of half-sib families $N\in \{10, 50, 100, 500\}$, the number of progeny in each half-sib family $n\in \{50, 100, 500, {1,000}\}$ and the number of SNPs $q\in \{{1,500}, {2,000}, {2,500}, {3,000}, {3,500}, {4,000}\}$. Here, $q={1,500}$ markers resembled a 50K SNP panel on the bovine genome and $q={4,000}$ reflected roughly a SNP density of 120K. In addition to the analysis of complete data sets, incompleteness was assumed in the $q={1,500}$ scenario and 50 % genotypes of progeny and parents were set randomly to unknown status.

*Semi-real population.* Complementing the synthetic simulation setup, meioses were simulated from empirical parental haplotypes. This procedure was exemplarily done for Brown Swiss cattle. All genotypes were phased and imputed towards the maximum density of 123K with FImpute v3 [49] by QUALITAS AG according to a protocol for Swiss breeding evaluations. Afterwards, SNPs on BTA4 and corresponding to “chip 4” were chosen ($q={2,403}$) and haplotypes of parents were extracted. (Based on the empirical data, no candidates of misplaced markers were situated on BTA4.) In total, 12 scenarios were generated: the number of half-sib families $N\in \{10, 50, 100, 500\}$ and the number of progeny per half-sib family $n\in \{50, 100, 500\}$. In total, 737 sires had more than 30 progeny in the imputed data set. Thus, *N* sires were randomly selected out of this set and mated at random with *n* out of 13,146 dams. We used a 1:1 transformation of physical distances in Mbp units into genetic distances in cM units. For simulating random crossovers between adjacent markers, the distance in M units were directly used as recombination rate disregarding any sex differences or crossover interference. Eventually, recombination events were estimated using the complete genotype information. For comparison, the analysis was repeated with 50 % genotypes of progeny and parents discarded at random.

*Evaluation of simulations.* Each simulation design was repeated 10 times and genetic coordinates were estimated by the HMM-based, the deterministic and the likelihood-based approach. Two quality measures were calculated in each repetition for male maps. Let $d_i$ denote the simulated genetic-map position of marker $i\in \{1,\ldots ,q\}$. First, accuracy (acc) of estimated map length was derived as

$$\begin{aligned} \text {acc}= & \widehat{d}_q / d_q. \end{aligned}$$

Second, mean squared error (mse) of estimated map positions $\widehat{d}_i$ of all markers $i=1,\ldots , q$ was determined as

$$\begin{aligned} \text {mse}= & \frac{1}{q}\sum _{i=1}^q\left( \widehat{d}_i - d_i\right) ^2. \end{aligned}$$

### Genomewide association study on recombination activity

Two recombination traits were investigated separately for sires and dams: autosomal crossover count and intra-chromosomal allelic shuffling. Crossover count was calculated as the average number of genomewide recombination events among progeny. Unlike crossover count, which reflects an absolute measure of recombination activity, shuffling gives a relative measure of recombination activity. Intra-chromosomal shuffling resembles the chance that an arbitrary pair of SNPs on the same chromosome recombines during meiosis [52]. It was determined from the estimated proportion of paternal ($p_{k,j(i)}$) and maternal ($1-p_{k,j(i)}$) chunks of chromosome *k* transmitted from parent *i* to its progeny *j*(*i*) as

$$\begin{aligned} \overline{r}_i= & \frac{1}{n_i}\sum _{j(i)=1}^{n_i}\sum _{k=1}^{29}2p_{k,j(i)}\left( 1-p_{k,j(i)}\right) L_k^2, \end{aligned}$$

with relative chromosome length $L_k$ and $n_i$ the number of progeny of parent *i*. For convenience, the position of recombination event (in bp units) was specified in the middle of a recombination interval, flanked by the nearest informative markers, obtained by the HMM-based approach. At first, sex- and breed-specific differences in recombination traits were verified via linear model analysis including fixed effects of sex, breed and interaction between sex and breed. The trait shuffling was centered and scaled over all data. Since there was a high variation in crossover count, especially with low number of progeny (see Additional File 1, Fig. S2-S3), progeny number was considered as weighting term. The significance of sex and breed was verified with a global *F* test from the analysis of variance. Afterwards, in case of significance, least-squares means per sex and per breed were determined, followed by a *t* test on pairwise differences of least-squares means using the R package emmeans v1.10.6 [31] in R v4.4.0 on Windows 10 x64. *P* values were adjusted by Tukey’s method to account for multiplicity.

At second, genomic best linear unbiased prediction (GBLUP) was performed separately for each breed, sex and recombination trait *y* to assess genomic heritability $h^2_g=\sigma _g^2/(\sigma _g^2+\sigma _e^2)$. The corresponding GBLUP model,

1 $$\begin{aligned} y= & \textbf{1}\mu +Zu+e, \end{aligned}$$

included $\mu$ the sub-population mean, *Z* the design matrix (identity matrix) for a random animal effect $u\sim N(0,G\sigma _g^2)$ of sires or dams with *G* the genomic relationship matrix of corresponding size and $e\sim N(0, D\sigma _e^2)$ the vector of residuals with *D* a diagonal matrix of reciprocal weights: $d_i = 1/n_i$ ($i=1,\ldots ,n_s$ for sires and $i=1,\ldots ,n_d$ for dams). The trait shuffling was centered and scaled. After filtering for the number of missing data on individual ($\le 20\,\%$) and on marker level ($\le 20\,\%$) with PLINK v1.9 [41], the *G* matrix was set up with use of the software GCTA v1.94.1 [56] and GBLUP was implemented with use of the software ASReml Release 4.2 [21]. Significance of the genomic variance component was tested via restricted likelihood ratio test with its null distribution being a 50:50 mixture of point mass at zero and $\chi ^2_1$ [50].

Additionally, a GBLUP model was applied to each breed and recombination trait comprising both sexes to inspect any cross-sex genetic correlation by specifying

2 $$\begin{aligned} y= & Wb +Zu+e, \end{aligned}$$

where *W* is the design matrix of fixed effects $b=(\mu , b_s, b_d)'$ with population mean $\mu$ and sex effects. Here, genetic effects *u* of dams and sires are assumed to be correlated,

$$\begin{aligned} u= & \left( \begin{array}{c}u_s\\ u_d\end{array}\right) \sim N(0, \Sigma \otimes G)\ \text {with}\ \Sigma =\left( \begin{array}{cc}\sigma _s^2& \sigma _{s,d}\\ \sigma _{s,d}& \sigma _d^2\end{array}\right) , \end{aligned}$$

with sire- and dam-specific genomic variance and covariance components.

At third, GWAS was carried out to identify genomic regions associated with recombination activity. To this end, the following linear mixed model was applied separately to each breed, sex and recombination trait,

3 $$\begin{aligned} y= & \textbf{1}\mu + Xm+Zu+e. \end{aligned}$$

The model components coincide with GBLUP model (1) and *X* is an $n_s\times 1$ design matrix for sires and $n_d\times 1$ design matrix for dams containing the parental genotype codes at the candidate SNP with additive effect *m*. Weighted GWAS was implemented with use of the software ASReml Release 4.2 [21]. Significance of association between a candidate SNP and phenotype *y* was tested by using the test statistic $T= \widehat{m} / \text {SE}(\widehat{m})$ and its approximation to a standard normally distributed random variable with distribution function $\Phi _{0,1}$. *P* values were obtained as

$$\begin{aligned} P= & 2\left[ 1- \Phi _{0,1}(|T|)\right] . \end{aligned}$$

Thresholds of $P=10^{-5}$ and $P=10^{-4}$ indicated strong and suggestive signals, respectively. In a distance of 500 kb upstream and downstream to a significant or suggestive SNP, information about nearest genes and their related gene function were examined via self-written R script. To this end, data from NCBI database https://www.ncbi.nlm.nih.gov/data-hub/gene/taxon/9913/ were downloaded (accessed on October 2, 2025). Furthermore, a list of recombination-related genes was compiled through a manual literature search following the keywords “recombination” and “meiosis” (see Additional File 2) and compared with the signals found.

## Results

### Recombination rate and genetic map

Beyond the summary of results given below, the essentially revised R Shiny app CLARITY v3.0.0 available via web interface at https://nmelzer.shinyapps.io/clarity provides supplemental information and illustrative figures for being interactively explored.

The HMM-based approach delivered information about intervals flanked by informative markers, where recombination events putatively happened during male and female meiosis. After filtering results of the first run for markers with MCS $\ge 0.986$ and progeny with $\le$58 crossovers (Table 2), recombination events were re-estimated based on 51,440 SNPs in Holstein-CH, 49,619 SNPs in Brown Swiss, 49,908 SNPs in Simmental, 50,992 SNPs in Original Braunvieh, 44,260 SNPs in Limousin and 40,973 SNPs in Angus. The number of markers shared between breeds is reported in Additional File 1 (Fig. S4). After the second LINKPHASE3 run, rare outliers with reduced MCS existed (minimum 0.926 in Angus) but in every breed 99 % of the SNPs had MCS $>0.986$. Due to the high degree of missing genotypes, not all genotyped animals provided sufficient information for crossover analysis. The number of genotyped animals, that were actually processed by the HMM-based approach, and the corresponding number of parents is given in Table 2. The average number of recombination events varied from 22.89 to 24.88 for sires and from 21.07 to 22.53 for dams among breeds (Table 3). Total male map length ranged from 26.97 M in Limousin to 29.83 M in Brown Swiss. Female map length was estimated in a range from 23.32 M in Limousin to 26.08 M in Angus. Female map length was estimated shorter than male map length; the difference was up to 18 % in Brown Swiss.

**Table 2:** Number of SNPs $q_\text {ex}$ and animals $n_\text {ex}$ removed before second run of HMM-based approach (run2), number of animals $n_{t,1}$ as input for run2, number of animals $n_{t,2}$ with detected crossover events as output from run2 and corresponding number of sires $n_{s,2}$ and dams $n_{d,2}$, number of sires $n_{s,3}$ and dams $n_{d,3}$ after filtering for missing data used in GWAS, number of SNPs $q_s$ and $q_d$ after filtering for missing data used in GWAS of sires and dams, respectively

Breed	$q_\text {ex}$	$n_\text {ex}$	$n_{t,1}$	$n_{t,2}$	$n_{s,2}$	$n_{d,2}$	$n_{s,3}$	$n_{d,3}$	$q_{s}$	$q_{d}$
Holstein-CH	163	37	76,838	62,672	2,487	15,501	671	1,946	51,380	51,603
BrownSwiss	261	21	68,029	60,351	2,346	11,358	836	1,143	49,520	49,875
Simmental	395	4	13,402	11,726	1,146	2,057	366	132	50,094	50,292
Original Braunvieh	260	5	12,599	11,211	1,065	2,377	427	1,051	50,750	50,987
Limousin	206	0	6,842	4,838	584	262	549	107	44,466	44,466
Angus	4,074	10	4,171	2,504	353	178	325	76	40,633	40,195

**Table 3:** Mean and standard deviation of recombination events of sires and dams, map length in Morgan units estimated by HMM-based, deterministic and likelihood-based approach based on male (m) and female (f) meioses

	HMM-based						Deterministic			Likelihood-based
Breed	mean f	SD f	Map length f	Mean m	SD m	Map length m	mean m	SD m	Map length m	Map length m
Holstein-CH	21.409	4.845	24.580	22.887	4.690	27.546	21.173	3.522	20.598	18.132
Brown Swiss	21.072	4.558	24.483	23.540	4.139	29.826	20.690	4.089	19.286	18.232
Simmental	22.394	4.872	24.306	23.902	5.134	29.246	22.946	2.528	22.422	12.087
Original Braunvieh	21.681	4.373	23.469	24.424	3.740	27.135	21.619	2.903	21.267	13.728
Limousin	22.058	4.258	23.319	24.880	4.113	26.971	24.594	2.264	24.517	12.511
Angus	22.527	5.676	26.078	24.658	5.394	28.662	23.514	1.722	23.437	9.317

**Fig. 1:**
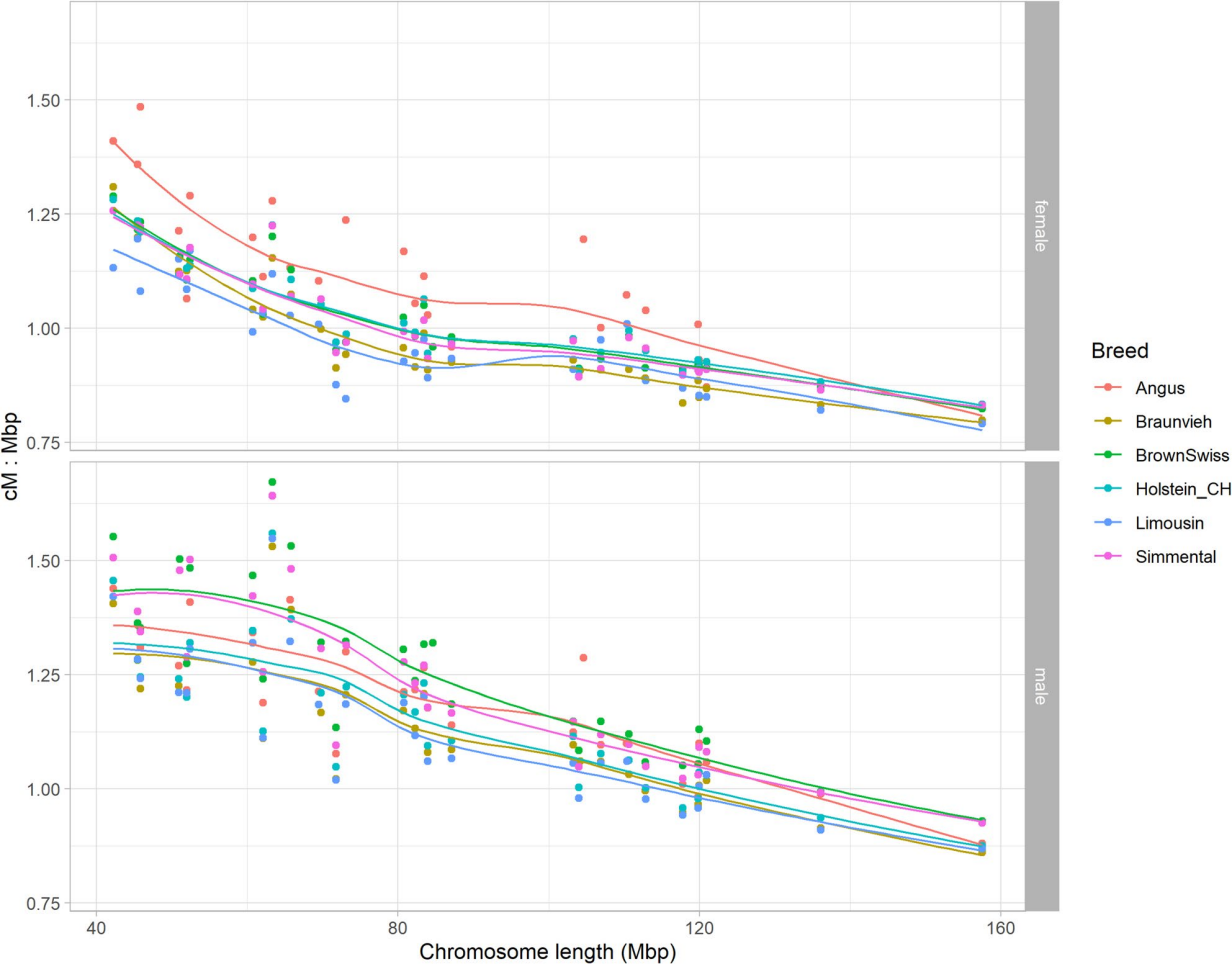
Ratio of chromosome length in cM derived from the HMM-based approach to length in Mbp versus total chromosome length

A chromosome-wise presentation of genetic vs. physical map coordinates obtained from the HMM-based approach is shown in Additional File 1 (Fig. S5 for male and Fig. S6 for female maps), often showing a mild S-shaped pattern of curves in males and a linear shape in females for all breeds. In dual-purpose breeds, male (female) genetic-map length of chromosomes was on average 1.7 % shorter and 1.6 % longer (2.4 and 3.1 % shorter) than in dairy and beef cattle, respectively. Strikingly, the length of female map in Angus exceeded other breed-specific female maps but this could be explained by the relatively small number of genotyped dams that received an estimate of crossover count ($n_{d,2}=178$, Tab. 2), leading to higher uncertainty in recombination rate estimates. Based on physical chromosome lengths according to ARS-UCD1.2, the cM:Mbp ratio varied from approximately 0.9 (all breeds on BTA1) to 1.7 (Brown Swiss on BTA19) in males, showing interaction with chromosome length (Fig. 1). The cM:Mbp ratio varied from approximately 0.8 (all breeds on BTA1) to 1.5 (Angus on BTA28) in females.

**Fig. 2:**
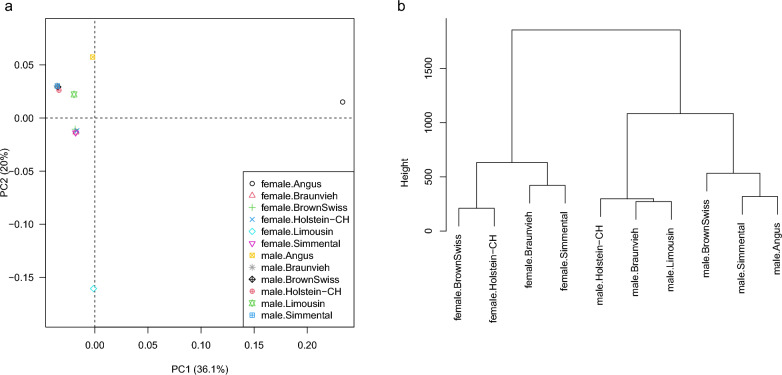
First versus second principal component of the recombination matrix (a) and dendogram cluster of genetic maps per sex and breed (b)

In total, 34,755 shared SNPs were considered for the analysis of similarity in terms of recombination rates between adjacent markers and genetic-map coordinates. Clustering of breeds through their representation of first and second principle components on recombination rates between adjacent markers (Fig. 2a) revealed two apparent clusters: (1) male recombination of Brown Swiss, Holstein-CH and Simmental and (2) female recombination of Brown Swiss, Holstein-CH, Simmental and Original Braunvieh. Recombination rates of female Angus and female Limousin deviated extremely from all other populations and these two female breeds were discarded from hierarchical clustering in the next step. The cluster dendogram is displayed in Fig. 2b. Male maps deviated clearly from female maps. Male maps of the trio Brown Swiss, Simmental and Angus clustered separately from the trio of male maps of Holstein-CH, Original Braunvieh and Limousin . Furthermore, female maps of dairy breeds Brown Swiss and Holstein-CH appeared closely related as well as female maps of dual-purpose breeds Original Braunvieh and Simmental.

Male map length obtained with the deterministic approach was up to 35 % shorter than with the HMM-based approach, see Table 3. The difference was even more extreme with results retrieved from the likelihood-based approach: map length was estimated up to 67 % shorter compared with the HMM-based approach. Curves derived from the likelihood-based approach, which revealed strong deviation between breeds depending on the population size, can be inspected in the CLARITY app. This outcome confirmed the need of criteria for evaluating the suitability of the estimation procedure used and particularly the role of genotype incompleteness.

### Simulation study

*Synthetic population.* With different simulated scenarios, accuracy of estimated male maps was evaluated and compared among methods. The genetic-map length was underestimated in general with the deterministic and the likelihood-based approach (acc $<1$) and slightly overestimated with the HMM-based approach (accuracy ranged from 1.01−1.05). The deterministic approach performed robustly in terms of accuracy (i.e. acc $>80\,\%$) for any population size and improved with increasing SNP density (Additional File 1, Fig. S7). The likelihood-based approach showed a strong dependence on both the number of families and the family size but was independent on marker density. It outperformed the deterministic approach if $N\ge 50$ and $n\ge 500$. If the population consisted of few families (here $N=10$), the likelihood-based approach required a correspondingly large number of progeny ($n={1,000}$) to perform better than the deterministic approach. Accuracy larger than 90 % was observed with the likelihood-based approach when $N\ge 50$ and $n={1,000}$ or $N\ge 100$ and $n\ge 500$. Remarkably, the HMM-based approach performed consistently better than the deterministic and the likelihood-based approach, independently of sample size and SNP density.

**Fig. 3:**
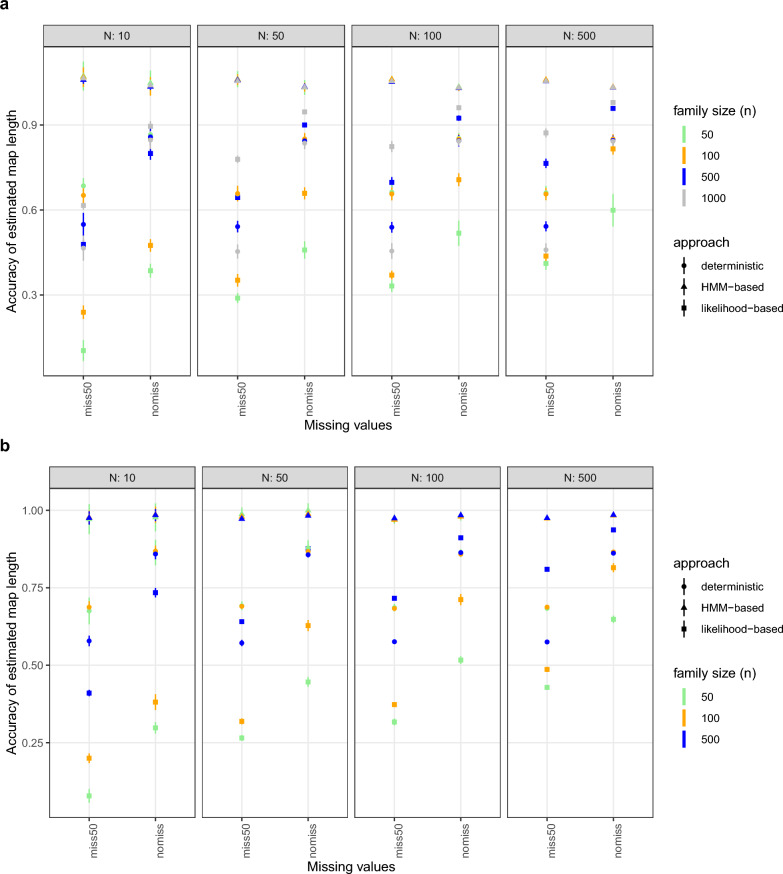
Accuracy of estimated male map length using the likelihood-based, the deterministic and the HMM-based approach in a simulation study based on (**a**) a synthetic population (1,500 SNPs) and (**b**) a semi-real population (2,403 SNPs) without (“nomiss”) or with 50 % (“miss50”) missing genotypes. Different panels refer to different numbers of half-sib families (*N*) used in the evaluation. The vertical coloured lines reflect the standard deviation of accuracy observed in 10 repetitions of simulation

Discarding 50 % genotypes at random slightly intensified overestimation of map length by the HMM-based approach (+2 % accuracy on average over 16 scenarios) and severely reduced accuracy of map length estimated by the deterministic and the likelihood-based approach by 32 % and 35 %, respectively, see Fig. 3a.

The quality measured in terms of mean squared error mirrored the observed accuracy, supporting all statements (Additional File 1, Fig. S7 and S8).

*Semi-real population.* Depicting the particular genomic architecture of Brown Swiss cattle with almost 50 % genotype information missing in the data set, a semi-real population should give further insight into the quality of estimated male genetic maps. Similarly to the conclusions drawn based on the synthetic population setup, the HMM-based approach performed best and yielded accuracy close to 1, irrespective of population size and presence of missing data, see Fig. 3b. Both the deterministic and the likelihood-based approach were not able to deal with the high amount of missing data and lost on average over the 12 scenarios 25 % and 40 % accuracy, respectively, compared to full data analysis. Again, mean squared error confirmed the superiority of the HMM-based approach, especially given the large amount of missing genotypes (Additional File 1, Fig. S8).

### Sex- and breed-specific differences in recombination activity

**Fig. 4:**
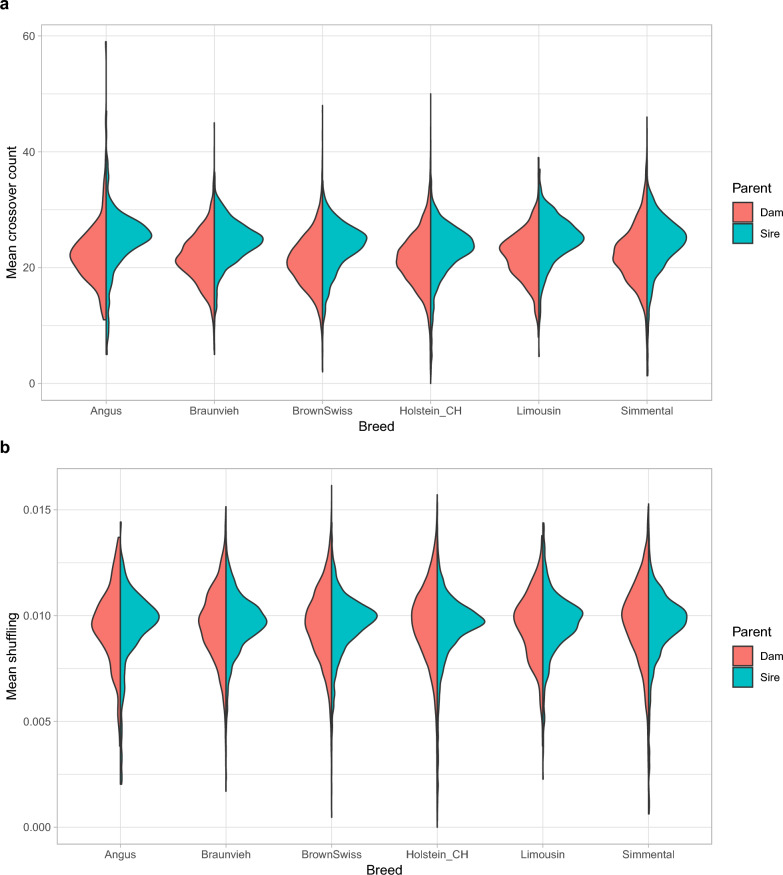
Distribution of mean parental autosomal crossover count (**a**) and intra-chromosomal allelic shuffling (**b**)

The distribution of autosomal crossover count and intra-chromosomal allelic shuffling grouped by sex and breed is displayed in Fig. 4 and Additional File 1 (Fig. S9 and S10). The variation of recombination traits, especially shuffling, was higher in females than in males; this outcome might be induced by the small number of genotyped progeny per dam in general. Also a matter of sample size, the traits were smoothly and symmetrically distributed only for dairy and dual-purpose breeds. The squared correlation between shuffling and crossover count ranged from 0.54 in Angus to 0.62 in Holstein-CH, Simmental and Limousin.

A linear model analysis revealed significantly different crossover counts between sexes as well as among breeds ($P<0.01$). The estimated effect size corrected for all other factors pointed out 2.60 (in Angus) to 4.67 (in Brown Swiss) more crossover events in sires than in dams. Derived from least-squares means, crossover count of sires differed significantly between all pairs of breeds ($P<0.01$) except Angus vs. Original Braunvieh, Angus vs. Limousin and Brown Swiss vs. Simmental. Significant differences of dam crossover count were only found between Angus vs. Brown Swiss, Angus vs. Holstein-CH, Original Braunvieh vs. Brown Swiss, Original Braunvieh vs. Simmental, Brown Swiss vs. Holstein-CH, Brown Swiss vs. Simmental and Holstein-CH vs. Simmental ($P<0.01$). Obtained from least-squares means, intra-chomosomal shuffling differed significantly between sires and dams within all breeds ($P<0.01$) except Angus. At most, shuffling in sires exceeded shuffling in dams by 0.23 phenotypic standard deviations in Brown Swiss. A comprehensive summary of linear model analysis is given in Additional File 3. Furthermore, sex- and breed-specific differences in recombination traits are visualised through least-squares means and their standard errors in Additional File 1 (Fig. S11-S12).

All GBLUP models were applied to genotype data after filtering for the proportion of missing genotypes on individual and marker level; the size of reduced data is provided in Table 2. Meaningful estimates of cross-sex genetic correlation via model (2) were obtained particularly for Holstein-CH (0.46 for crossover count, 0.59 for shuffling), Brown Swiss (0.57 for crossover count, 0.72 for shuffling) and Original Braunvieh (0.32 for crossover count, 0.13 for shuffling), see Additional File 3. These estimates clearly showed that recombination traits are distinct characteristics between sires and dams. Unfortunately, REML could not converge within 100 iterations in the smaller populations. In breed- and sex-specific GBLUP analyses via model (1), genomic variation was found to be significantly present in all breeds and sexes ($P<0.01$) except Brown Swiss female shuffling, Angus male shuffling, Angus female crossover count and shuffling, Limousin female crossover count and shuffling (see Additional File 3). In case of significance, estimates of genomic heritability of crossover count varied from 0.06 in Holstein-CH sires to 0.21 in Simmental sires and from 0.07 in Brown Swiss dams to 0.50 in Simmental dams. Estimates of genomic heritability of shuffling ranged from 0.02 in Holstein-CH sires to 0.04 in Brown Swiss sires and from 0.06 in Holstein-CH dams to 0.45 in Simmental dams.

**Fig. 5:**
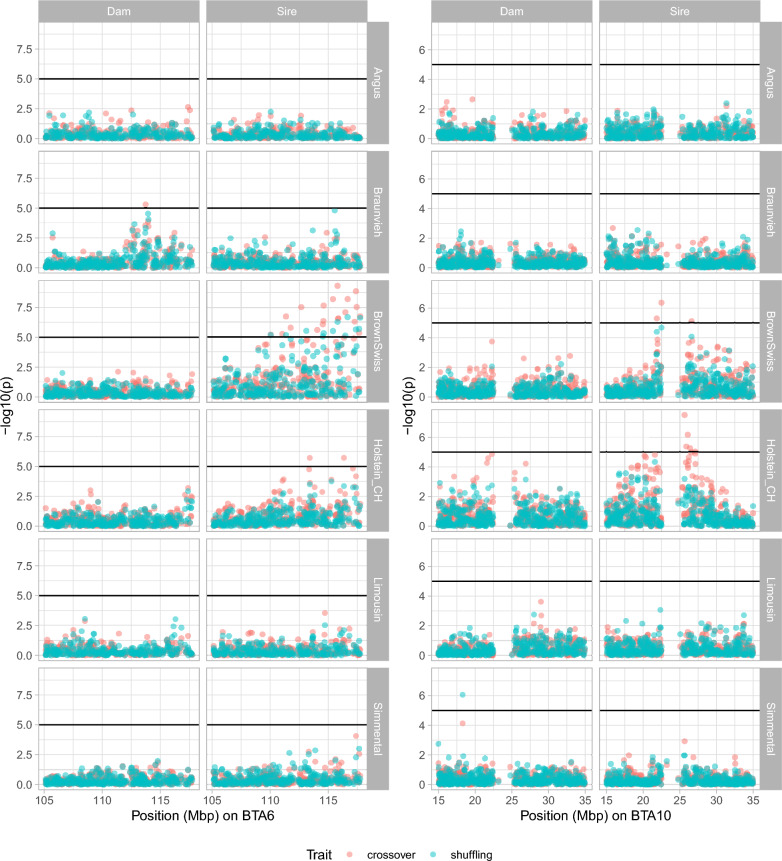
Manhattan plot of GWAS results on autosomal crossover count and intra-chromosomal allelic shuffling per breed and sex around two candidate regions: BTA6: 105-118 Mbp (left) and BTA10: 15-35 Mbp (right), horizontal line corresponds to threshold $P=10^{-5}$

The study of putative associations of single SNPs with autosomal crossover count or intra-chromosomal allelic shuffling per parent (model 3) revealed strong indications seen as intervals of elevated $-\log (P)$ values particularly in Brown Swiss and Holstein-CH. In total, 46 candidate loci with $P<10^{-5}$ were detected in these breeds: 34 SNPs were linked to crossover count and 12 SNPs were associated with shuffling, pointing to two chunks of chromosomes on BTA6 and BTA10 (Fig. 5). Regions supported by $\ge 3$ strong or suggestive signals are listed in Table 4. On BTA6 within 108.99-−117.74 Mbp, strong impact on both sire crossover count and sire shuffling was found in Brown Swiss ($P\ge {4.86}\cdot 10^{-10}$ and $P\ge {2.03}\cdot 10^{-7}$, respectively) and on sire crossover count in Holstein-CH ($P\ge {1.90}\cdot 10^{-6}$). The region 19.27-−27.13 Mbp on BTA10 was identified for crossover count in Holstein-CH sires ($P\ge {3.09}\cdot 10^{-8}$) and dams ($P\ge {1.36}\cdot 10^{-5}$); it was also found in Brown Swiss sires ($P\ge {4.31}\cdot 10^{-7}$). This chunk was suggestive for shuffling in Brown Swiss sires ($P\ge 2.13\cdot 10^{-5}$). Another suggestive signal appeared in Holstein-CH sires at the end of BTA10 around 86.26 Mbp with impact on crossover count ($P {\ge 1.51}\cdot 10^{-5}$). The region 50.83-−54.05 Mbp on BTA18 was suggestive for crossover count in Holstein-CH sires ($P\ge 2.83\cdot 10^{-5}$). If crossover count was used as an additional covariate in the association analysis of shuffling, no signals were retained. GWAS in Original Braunvieh and Simmental revealed further spurious signals but no clear indication. Manhattan plots are provided for all cattle breeds in Additional File 1 (Fig. S13-S18).

In the proximity of $\pm 500$ kb of strong signals ($P<10^{-5}$) for crossover count and shuffling, a set of genes with known association to recombination activity were located: *RNF212*, *GAK*, *PCGF3* on BTA6 and *RNF212B* on BTA10. Significant SNPs being close to *RNF212B* were also in strong LD with *REC8* ($r^2>0.3$). In the vicinity of the suggestive signals on BTA10, *MLH3* and *NEK9* were situated. An overview of SNPs with $P<10^{-4}$, complemented by Benjamini-Hochberg corrected *P* values, and their nearest genes are reported in Additional File 4 for breed/sex/trait combinations with significant genomic variation.

**Table 4:** GWAS summary about chromosome regions associated with crossover count (nco) or shuffling (shuff); begin and end of region in Mbp with number of SNPs included, variance explained by SNPs in the given interval was roughly approximated as $\sigma _x^2=\sum _i 2f_i(1-f_i)m_i^2$ with effect size $m_i$ according to model (3) and allele frequency $f_i$ of SNP *i*, minimum *P* value observed

Chr	Breed	Parent	Trait	Begin	End	SNPs	$\sigma _x^2$	*P*
6	Brown Swiss	Sire	nco	108.988	117.745	32	8.063	4.86 · 10^−10^
6	Brown Swiss	Sire	shuff	110.045	117.745	20	0.213	2.03 · 10^−7^
6	Holstein-CH	Sire	nco	113.356	117.390	5	1.114	1.90 · 10^−6^
10	Brown Swiss	Sire	nco	21.862	26.605	3	0.796	4.31 · 10^−7^
10	Brown Swiss	Sire	shuff	21.862	26.605	3	0.030	2.13 · 10^−5^
10	Holstein-CH	Dam	nco	21.604	26.892	4	1.030	1.36 · 10^−5^
10	Holstein-CH	Sire	nco	19.265	27.128	18	4.114	3.09 · 10^−8^
10	Holstein-CH	Sire	nco	86.260	86.323	4	0.758	1.51 · 10^−5^
18	Holstein-CH	Sire	nco	50.833	54.054	3	0.538	2.83 · 10^−5^

## Discussion

In this study, genetic maps were derived for six European cattle breeds. An additional, but in this context absolutely necessary simulation study gave insight into the accuracy of maps obtained with varying population sizes. Hence, the use of the two comparative methods, the deterministic and the likelihood-based approach, cannot be recommended for empirical settings where (1) half-sib family size, (2) number of half-sib families and, most importantly, (3) amount of missing data are challenging factors. The strongest benefit of the superior method, the HMM-based approach, was its robustness in terms of all three factors. Furthermore, it allowed deriving sex-specific recombination rates. Total male map length differed up to 2.86 M and female map length differed up to 2.76 M among breeds. Chromosomes BTA6 and BTA10 were outstanding for their obvious associations with crossover count and intra-chromosomal shuffling in sires, where BTA10 also played a role in crossover count in dams.

### Accuracy of genetic maps

The number (*N*) of paternal half-sib families and their average size ($\overline{n}$) strongly varied in the underlying data sets, from $N={1,067}$ families with $\overline{n}=4$ progeny in Angus to $N={4,891}$ with $\overline{n}=16$ in Holstein-CH, see Sect. 2. These population structures differed to earlier studies where $N={1,053}$ families with $\overline{n}=349$ progeny were included in Holstein-DE analysis [42] and $N=6,866$ families with $\overline{n}=44$ were considered in German/Austrian Fleckvieh [35]. Correspondingly, methods exploiting linkage information within half-sib family were favoured in those studies. In contrast to recent investigations, Holstein-DE and Fleckvieh cattle were solely genotyped with the Illumina Bovine SNP50 genotyping array. Thus, the present study was also committed to explore sample size limitations required for estimating a genetic map with high accuracy and to assess the impact of genotype incompleteness. In the simulation setup, *N* and *n* were specified to also cover the population structure of Holstein-DE and Fleckvieh, confirming the high quality of maps obtained earlier. While rarely missing genotypes in Holstein-DE and Fleckvieh were imputed during preprocessing of the data, this step was skipped for the Swiss cattle populations. The gaps due to systematically missing data were rather large for imputation attempts, which could have introduced overrepresented haplotypes. For instance, on BTA1 in Simmental cattle, most frequently a region with 15 SNPs spanning 845 kb was missing in 27 % of the samples but also longer chunks of 69,242 kb covering 1,328 SNPs were missing in 2 % of the samples. In the simulation study, genotypes were discarded randomly instead of systematically. Nevertheless, the outcome impressively showed that genotype incompleteness did not affect the accuracy of map length estimated by the HMM-based approach. Although it must be admitted, that the HMM-based approach took the available parental genotype information into account, whereas the deterministic and likelihood-based approach solely employed the progeny genotypes.    

The likelihood-based approach remains a useful alternative for populations consisting of huge half-sib families with fully genotyped progeny and, regardless of sample size, for the detection of markers that are putatively misplaced in the underlying genome assembly: the occurrence of unusually high recombination rates between pairs of SNPs determines a candidate marker. All candidates of misplacement found in this study were among those SNPs with MCS below the threshold and were removed from genetic-map estimation. A breed-specific list of putatively misplaced markers (about 37–52 per breed) is available from the CLARITY app.

Another factor that may influence the accuracy of genetic maps is the occurrence of near double crossovers. In beef cattle, for instance, a double crossover within $<2$ Mbp distance was declared conspicuous and might occur from phasing errors [53]. In the literature, thresholds were chosen dependent on species and ranged from e.g. 1 Mbp in human to 3 Mbp in sheep [10, 40]. The distance of markers to telomere end (where increased recombination is expected; [38]) and the strength of crossover interference [8] influence the chance of near double crossovers. Exclusion of these rare events may further reduce the bias of estimated genetic-map coordinates. In our study, the proportion of near double crossovers to total number of crossovers per chromosome was on average less than 0.9 % for all breeds. This proportion was larger in synthetic populations ($<1.8$ %, with crossover interference) and semi-real populations ($<2.5$ %, no crossover interference) when 50 % genotypes were missing. In both simulation settings, the accuracy of estimated genetic map length was close to one and we neglected further cleaning steps for the empirical cattle data. Genotyping and phasing errors were taken into account on marker and individual level by filtering for MCS $\ge 0.986$ and $\le 58$ crossovers in general, thus minimising disrupting influences during genetic-map estimation.

### Sex- and breed-specific recombination

Recombination activity in terms of crossover count and intra-chromosomal shuffling was consistently higher in sires than in dams in all breeds with outstanding observation in Brown Swiss cattle (see Sect. 4). Correspondingly, male map lengths were longer than length of female maps with maximum difference of 5.34 M in Brown Swiss. Similar outcome was observed in Fleckvieh cattle where the male map exceeded the female map by 2.89 M [1]; the difference in Norwegian Red cattle was 1.89 M [6]. Not only in cattle, but also in other mammals, such as sheep [25], longer male maps were observed. Because longer female maps were found, for instance, in pigs [23], higher recombination rate cannot be attributed to a particular sex in general [5]. Variation in sex differences in recombination is widespread in nature, but there is no comprehensive explanation of its causes [48] and molecular mechanisms underlying the different genetic architectures are still to be determined [24].

Out of 28 recombination-related genes retrieved from literature (Additional File 2), four regions with strong signal were found in our study: *GAK*, *PCGF3*, *RNF212* and *REC8*/*RNF212B*. Most frequently in the literature, associations between crossover count and *RNF212* or its paralog *RNF212B* were observed for a range of species, e.g. cattle [27, 47], pigs [7], sheep [25], red deer [26] and human [28]. Influence of *GAK* and *PCGF3* on recombination was found in sheep [25]. Beside these well established regions, we found association between recombination activity and *POLN* being in close neighbourhood to *RNF212*. The protein encoded by *POLN* is involved in DNA repair and homologous recombination [36]. Among suggestive signals, we identified *NIPBL* which plays a role in meiotic prophase in mice [29]. Though more genes are listed in Additional File 4, they occurred as standalone signals and were not further verified. A strong association found in pigs between intra-chromosomal shuffling and the hotspot positioning gene *PRDM9* [7] did not show up in the cattle data sets.

In the underlying study, high similarity appeared between Brown Swiss and Simmental male genetic maps. Though there was rare exchange of genetics between these breeds, i.e. 0.15 % of Brown Swiss animals have Simmental ancestors, the impact on genetic-map estimation was assumed to be negligible. Discarding sex, a phylogenetic similarity between these two breeds has also been reported by Decker et al. [12]. We found that Original Braunvieh male maps were more closely related with Limousin and Holstein-CH than with Brown Swiss. Although Brown Swiss’ demographic history traces back to Original Braunvieh founders, they have been identified as genetically distinct populations taking samples of male genotypes into account [51] – now this separation was also obvious from their genetic maps. It would be interesting to consider additional measures of similarity between breeds and investigate, for instance, haplotypes shared in *RNF212* and *RNF212B* regions in future studies. Moreover, also in line with our results, the study of population structure revealed the largest genetic distance between Brown Swiss and Holstein-CH populations [34, 51]; this outcome was based on male data only. Since we found that female genetic maps were clearly distinct from male maps and Brown Swiss was connected to Holstein-CH from the female-map perspective, it would be highly informative to consider sex differences in future population structure analysis.

Estimates of heritability varied strongly between breeds, especially for female crossover count: from $h^2=0.07$ (SE 0.03) in Brown Swiss dams to $h^2=0.50$ (SE 0.16) in Simmental dams. For various cattle breeds, heritability estimates have been reported in a range between 0.04 and 0.26 depending on sex and statistical model [6, 47, 53]. Pedigree-based relatedness was imperceptibly low with an average of 0.09 among Simmental dams. For the breeds under investigation, the high amount of missing genotype data constituted a particular challenge for the detection of crossover events. However, since the proportion of missing data was similar between Simmental (e.g. on average 44 % on BTA1; Additional File 1, Fig. S1) and Brown Swiss (48 % on BTA1), sample size was probably the driving force: estimation of $h^2$ was based on $n=132$ Simmental dams and $n={1,143}$ in Brown Swiss dams. Populations with even lower sample size, i.e. Angus and Limousin dams (Tab. 2), did not reveal significantly present genetic variation (Additional File 3). For a rough assessment of sample size required, the length of confidence interval outlines the precision of variance component estimate. Based on an approximate chisquare distribution assumption, $n\ge 200$ is recommended to obtain a 99 % confidence interval half as long as the estimate of variance component itself. Consequently, the heritability estimate obtained in Simmental should not be overemphasised.

### Genome assembly

For the standardised analysis workflow used, physical coordinates of SNPs were taken from Hereford’s ARS-UCD1.2 genome assembly as a reference. However, breed-specific genome assemblies could have been included instead. For instance, genome assemblies of New Zealand Holstein (BioProject accession PRJNA668863), Simmental (PRJNA677947) and Angus (PRJNA432857) as well as Original Braunvieh and Brown Swiss [32] are available. Although the total physical map length was similar for these breeds ranging from 2,468,16 Mbp to 2,617,26 Mbp, huge deviations to the reference genome existed on a chromosome-wise level. As extreme examples, Angus BTA28 was 21 % shorter and Brown Swiss BTA22 was 22 % longer than corresponding chromosomes in ARS-UCD1.2 (Additional File 1, Fig. S19). Least deviations were observed for Simmental. Furthermore, some chunks of DNA were assigned to other chromosomes. For instance, the probe sequence of SNP BovineHD0100027042 was found on BTA23 in Simmental instead of BTA1 in Hereford using Blast (https://blast.ncbi.nlm.nih.gov/, March 14, 2024). The physical coordinates of SNPs are required for ordering them prior to estimating genetic-map coordinates. In this study, all markers with reduced MCS based on ARS-UCD1.2 were discarded. With a breed-specific physical map, the number of markers with reduced MCS, pointing to genotyping and phasing errors but also to potentially misplaced markers in the reference genome, probably decreases. Eventually, denser and more accurate genetic marker maps may be obtained.

### Selection signatures

The likelihood-based approach provides pairwise estimates of recombination rate which can be displayed as a kind of correlation plot. Bands of missing values may occur if sires are homozygous at one or both markers under inspection. Those bands help revealing/confirming selection signatures. For instance, Bhati et al. [2] identified a region of selection signature on BTA11 using composite likelihood ratio test (at about 66.0−68.5 Mbp) or iHS statistic (at about 68.4−69.2 Mbp) in Original Braunvieh. This region was also visible from the recombination rate plot as band of missing values due to homozygosity of sires in Original Braunvieh and does not show up in other cattle breeds. For instance, Additional File 1 (Fig. S20) displays this region in Original Braunvieh (left panel) and Brown Swiss (right panel). Note that 46 out of 133 SNPs within 66.0−69.2 Mbp did not pass the initial MAF filtering step based on all genotyped animals in our study.

A thorough selection signature analysis for the included Swiss cattle breeds is underway where a potential benefit of including breed- and sex-specific genetic maps will be assessed. Such kind of analysis will give further insight into shared demographic history.

### Archive of maps

The CLARITY app has been updated to contain estimated genetic maps, recombination rates between adjacent SNPs and summary statistics for eight European cattle breeds in total. Previous results obtained for Holstein-DE [42] and Fleckvieh [35] are now complemented by recent results based on the Swiss breeds. Especially, the use of the likelihood-based approach requires a sufficiently large population to reach accuracy of estimated map length of at least $80\,\%$. The larger the families, the better the quality of estimated map length and map positions. The inferences drawn from the simulation study led to the development and implementation of a colour score called “likelihood quality signal” in the CLARITY app. Like a traffic light, green colour is indicating that the likelihood-based approach performs with high quality and is superior to the deterministic approach (i.e. $N\ge 50$ and $n\ge 500$ or $N\ge 10$ and $n\ge {1,000}$), orange colour is used if the population size is sufficient to reach accuracy $\ge 80\,\%$ (i.e. $N\ge 500$ and $n\ge 100$). In all other cases, red colour represents that the likelihood-based approach will yield a low-quality genetic map. With this signalling, the archive of maps transparently combines maps estimated with the likelihood-based approach for all cattle populations. Genetic maps of this archive are offered to be used in several fields of genome-based evaluation, such as improving haplotype phasing (e.g. [13, 39]) and implementing novel breeding strategies that rely on Mendelian sampling variance (e.g. [4, 30, 37]). To fully exploit this archive, software tools must be enhanced to enable imputation and phasing techniques with respect to sex-specific maps.

## Conclusions

The present comparative study gave insight into sex- and breed specific differences in recombination activity and resulting genetic maps in different European cattle breeds. Three approaches were used for genetic-map estimation, an HMM-based, a deterministic and a likelihood-based method. Only the HMM-based approach was able to deal with the relatively high amount of systematically missing genotypes in the underlying data sets, which was due to the nature of local data acquisition, and provided estimates of male and female map of high accuracy. Breed-specific female maps could be clearly differentiated from male maps. Moreover, female maps of dual-purpose breeds as well as female maps of dairy breeds clustered together suggesting a closely connected demographic development. A differentiation of male maps with respect to breed purpose was not given. We have outlined several applications of sex-specific maps to further enhance computational work in animal breeding.

## Additional file

Supplementary material 1. PDF file with all supplemental figures

Supplementary material 2. XLSX file containing a list of known genes related to recombination activity

Supplementary material 3. XLSX file containing results of linear model analysis of recombination traits: autosomal crossover count (nco) and intra-chromosomal allelic shuffling (shuff); size -- effect size *m* according to model (1), frq -- minor allele frequency, var.exp -- variance explained by SNP determined as 2frq(1-frq)*m*^2^

Supplementary material 4. XLSX file containing information about SNPs associated with autosomal crossover count (nco) or intra-chromosomal allelic shuffling (shuff) (p<10^-4^)

## Data Availability

Restrictions apply to the availability of the original data supporting the findings of this study due to third party ownership. Genotype data are available from Qualitas AG (Zug) upon agreement. The R package CLARITY v3.0.0 including the source code (DOI 10.5281/zenodo.18668018 ) and processed data (DOI 10.5281/zenodo.17909700) on recombination rates and genetic maps can be downloaded from https://github.com/nmelzer/CLARITY under the terms of GPL ($\ge$ v2.0) and CC BY 4.0. Data on autosomal crossover count and intra-chromosomal allelic shuffling as well as R scripts for GBLUP and GWAS workflows are available at DOI 10.5281/zenodo.17951753 (CC BY 4.0 and GPL ≥ v3.0) https://zenodo.org/records/17951753?preview=1%20token=eyJhbGciOiJIUzUxMiJ9.eyJpZCI6IjM0YjI3NjgyLWM4MGMtNDc3ZC04YTE1LWU1Njg3Yzc1MTIwMiIsImRhdGEiOnt9LCJyYW5kb20iOiJiNGU4MzFhNzRkN2RlZGZiNjQ2N2Y1NzExOTY2ODRiNCJ9.Xnhb1VuDebmwa78wXZdOR163mD_VdrPIY3lul9ucQDrjQ0DeR8qoZs3lsRzFSOQdfJPlBS9-o27augMhQqp5VQ.
