# Supplementary material for: A comparative study on recombination activity in cattle
Source: Genet Sel Evol. 2026 Apr 6;58:24. doi: 10.1186/s12711-026-01041-0 (PMC13067647; doi:10.1186/s12711-026-01041-0)
Supplement: Supplementary file 1 — Supplementary material 1. PDF file with all supplemental figures [file 12711_2026_1041_MOESM1_ESM.pdf]

Supplemental figures to publication

A comparative study on recombination activity in cattle

by Wittenburg *et al.*

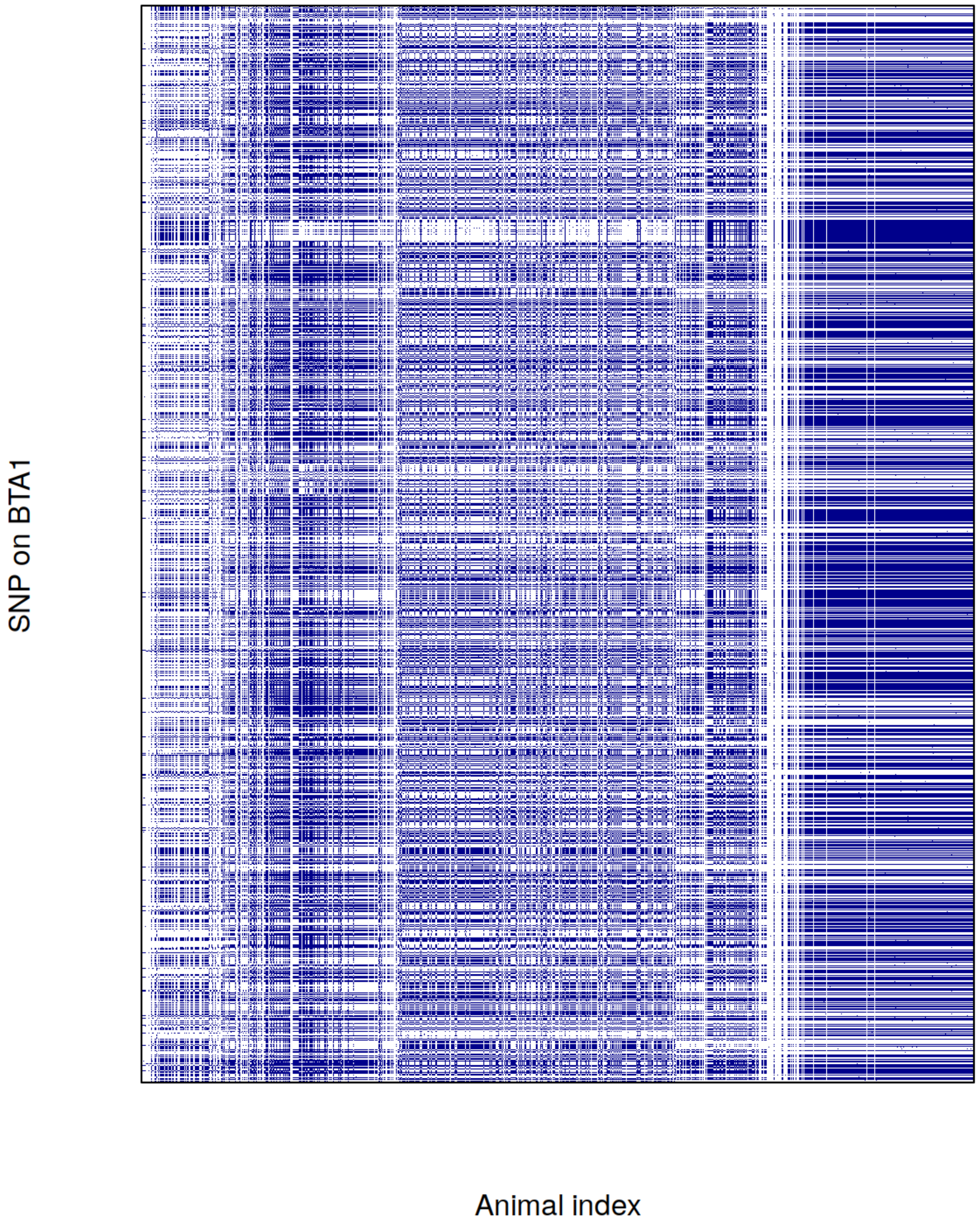

Figure S1: Visualisation of missing genotypes (in blue; in total 44 %) on BTA1 in Simmental.

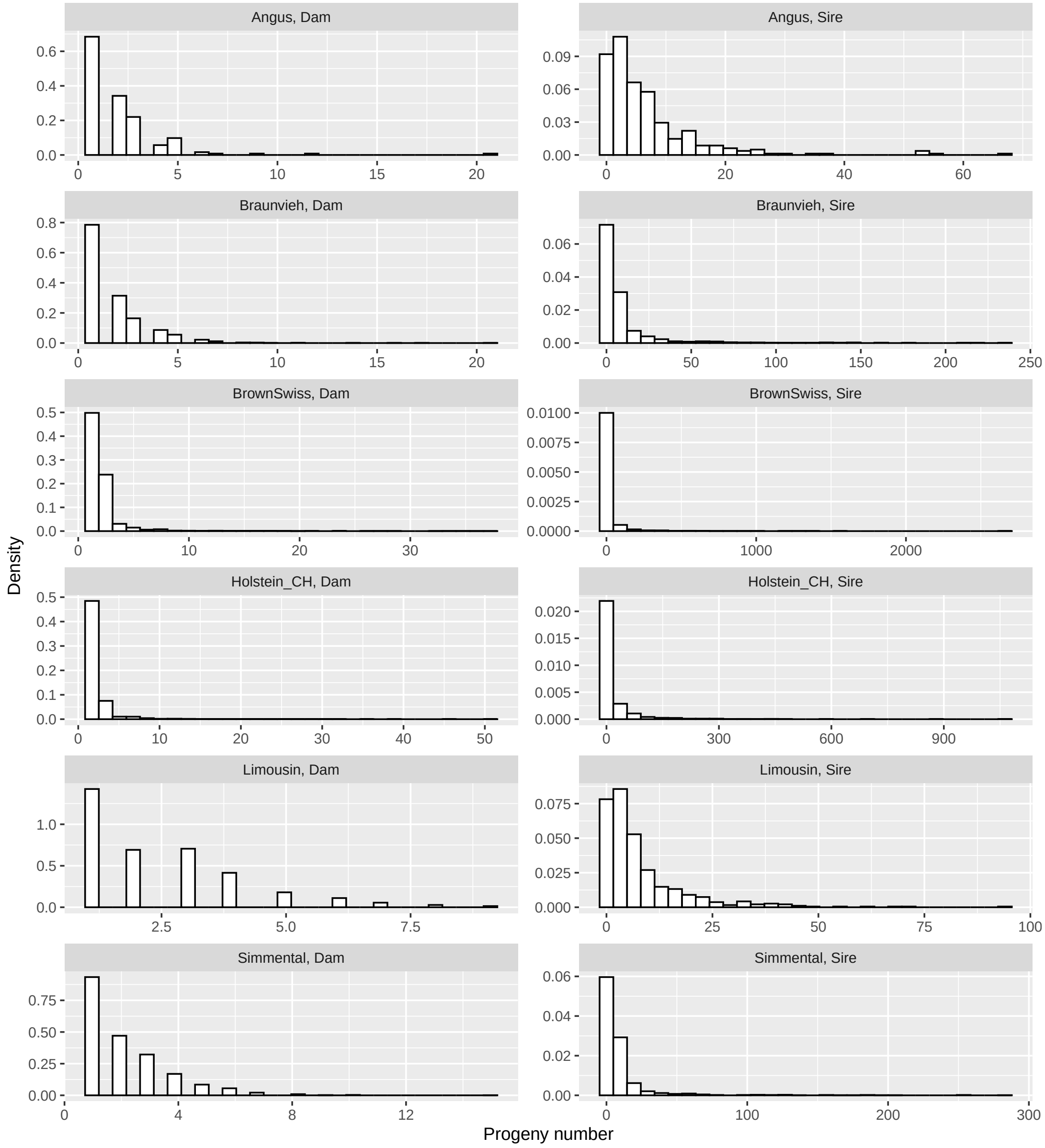

Figure S2: Histogram of progeny number (= weight in GWAS) per parent and breed; only parents with estimated autosomal crossover count were considered.

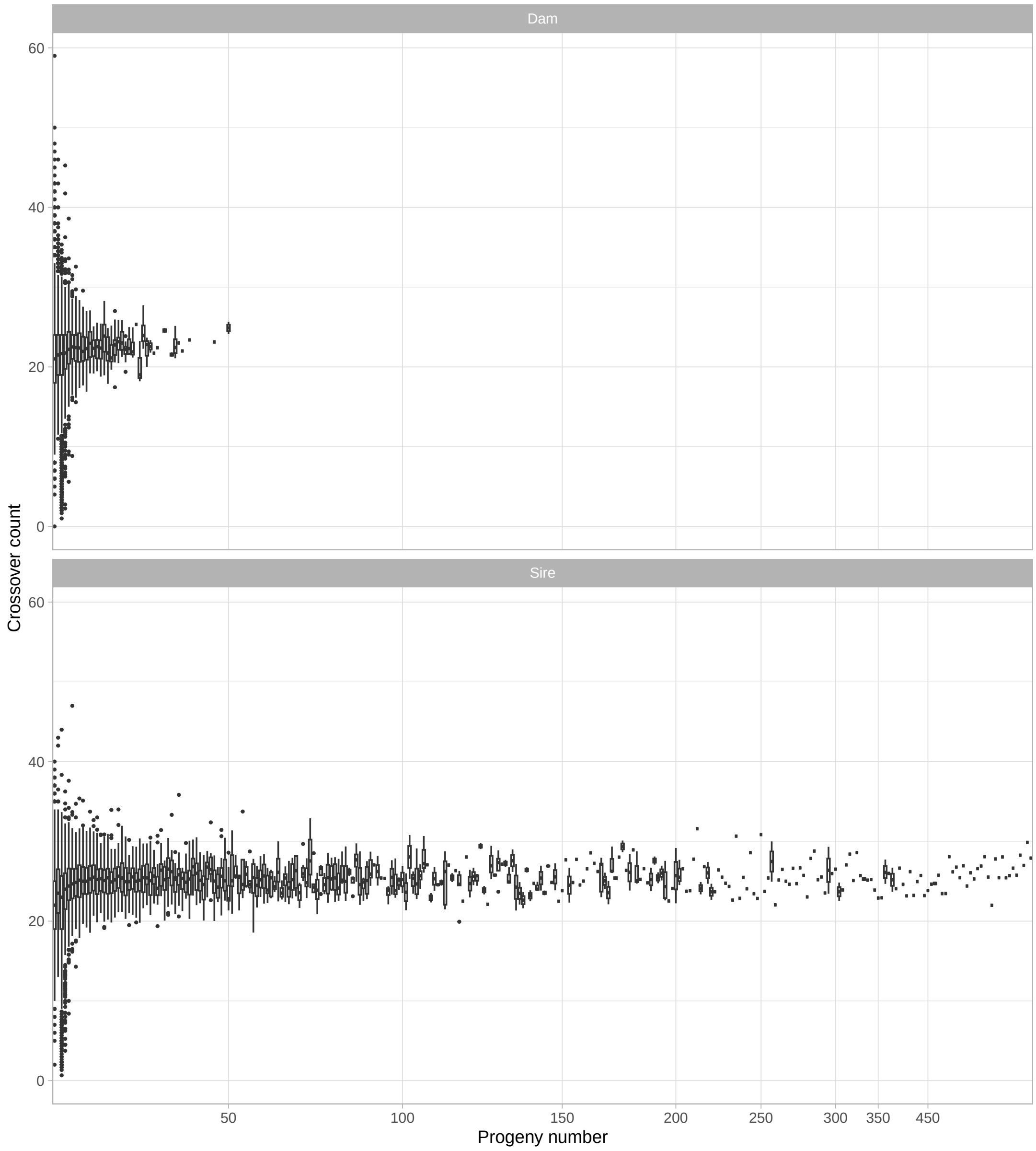

Figure S3: Boxplots of autosomal crossover count depending on progeny number over all breeds.

	BrownSwiss	Braunvieh	Holstein_CH	Simmental	Angus	Limousin
BrownSwiss	49619	48833	47002	46511	37162	40503
Braunvieh	48833	50922	48128	47527	38101	41487
Holstein_CH	47002	48128	51440	49203	38713	41869
Simmental	46511	47527	49203	49908	37763	41106
Angus	37162	38101	38713	37763	40973	39980
Limousin	40503	41487	41869	41106	39980	44260

Figure S4: Markers (used in LINKPHASE3 run2) shared between cattle breeds.

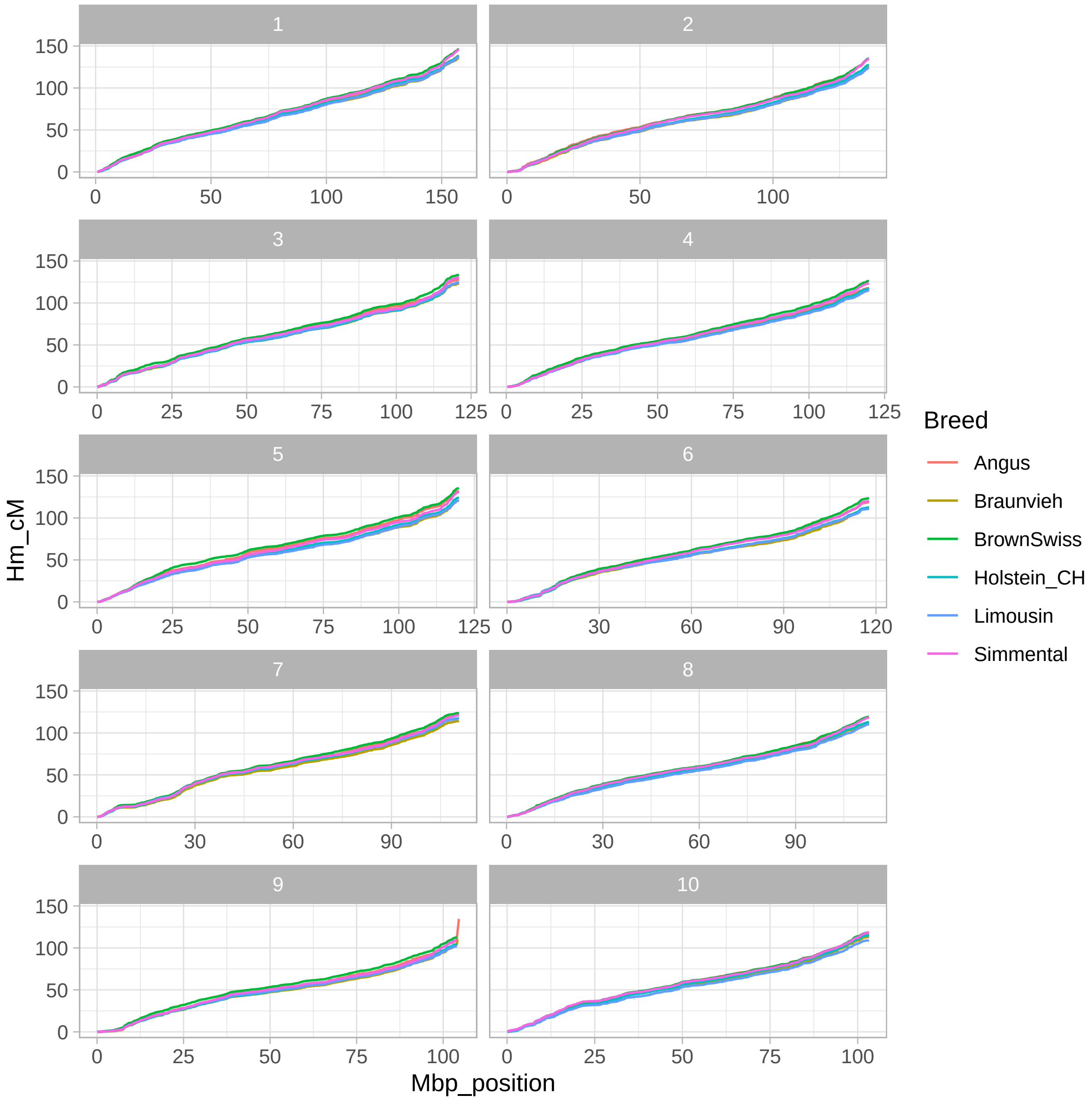

Figure S5: Male genetic map for every chromosome derived from the HMM-based approach for 6 cattle breeds.

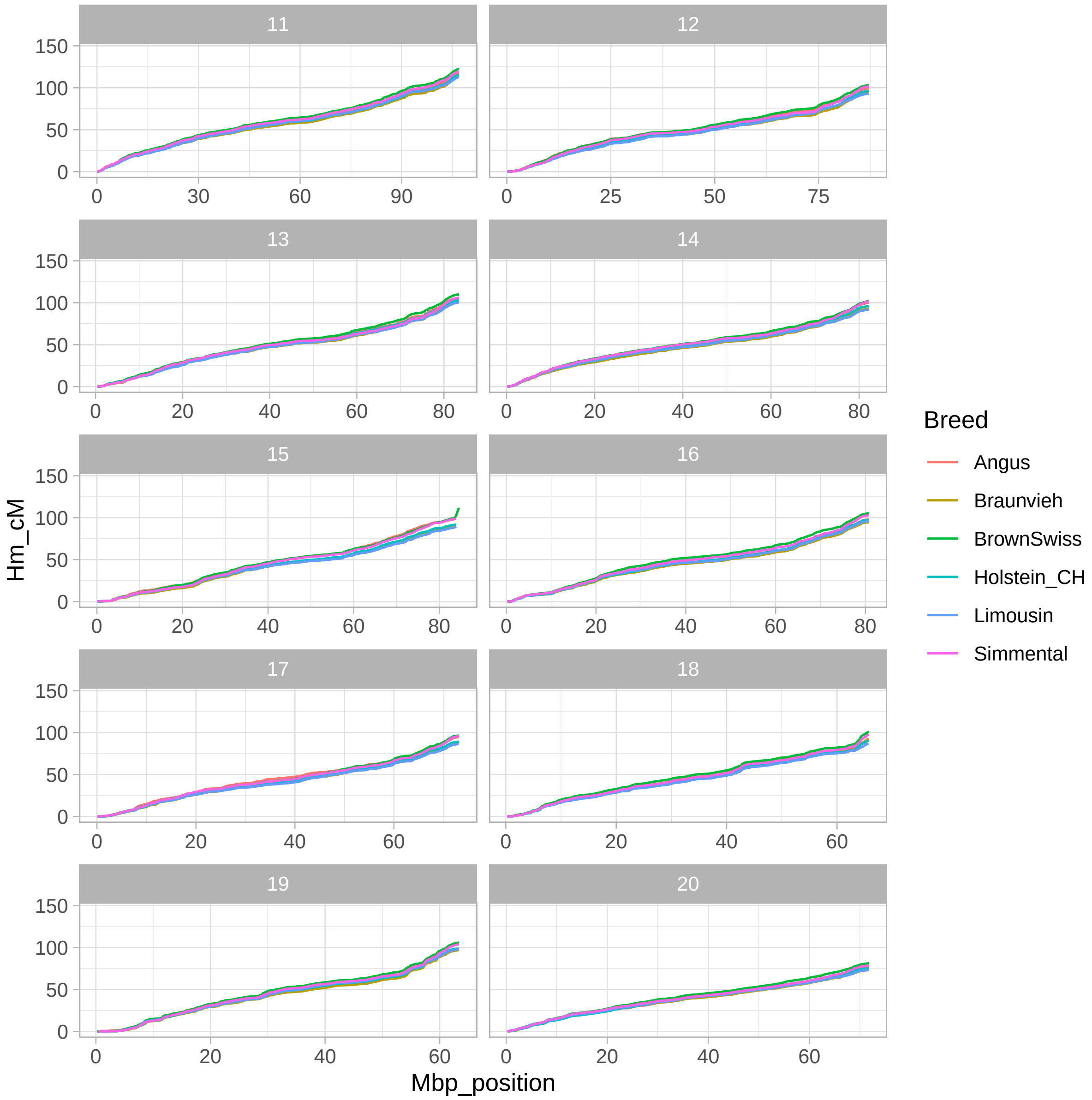

Figure S5: (cont.)

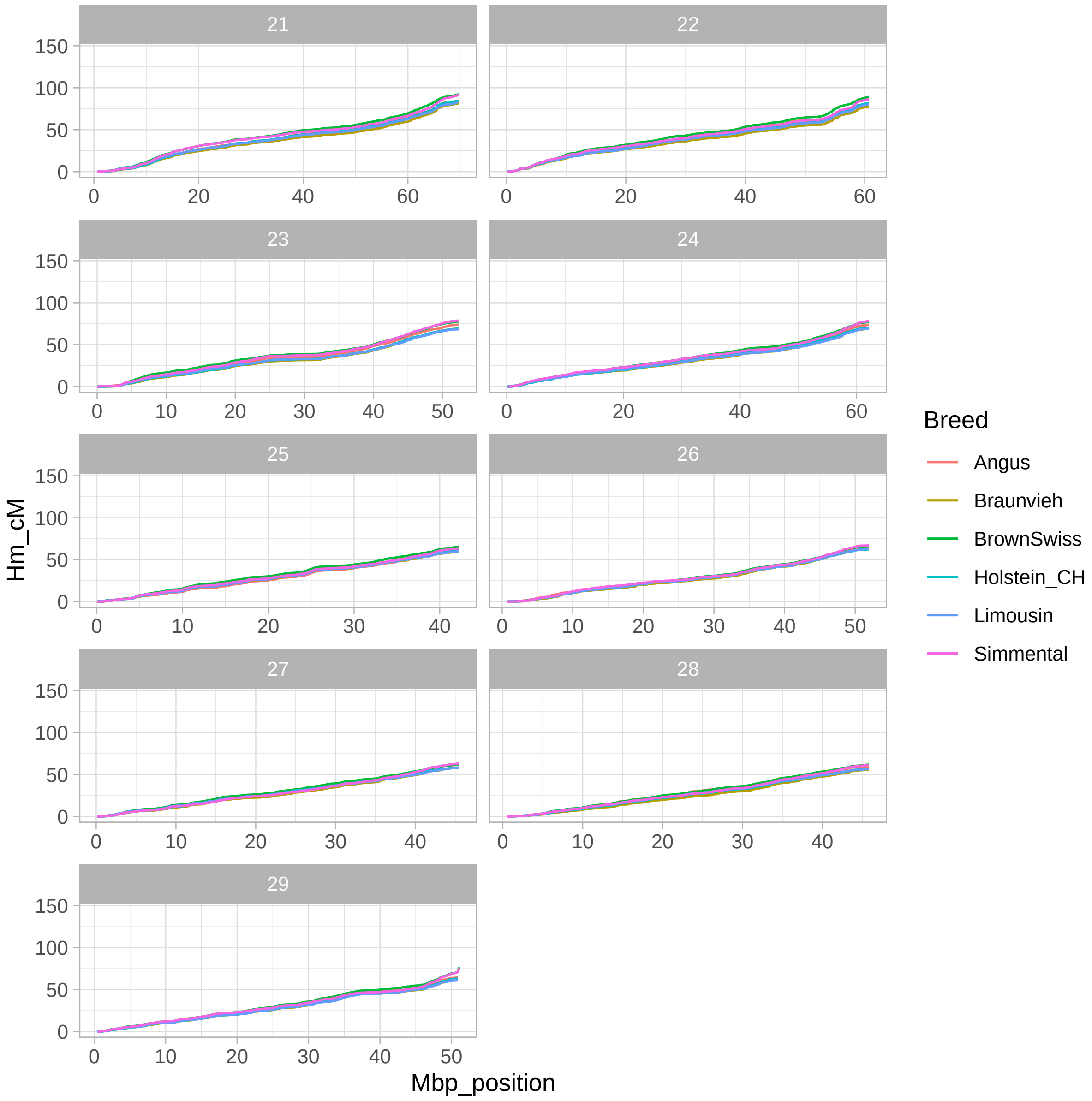

Figure S5: (cont.)

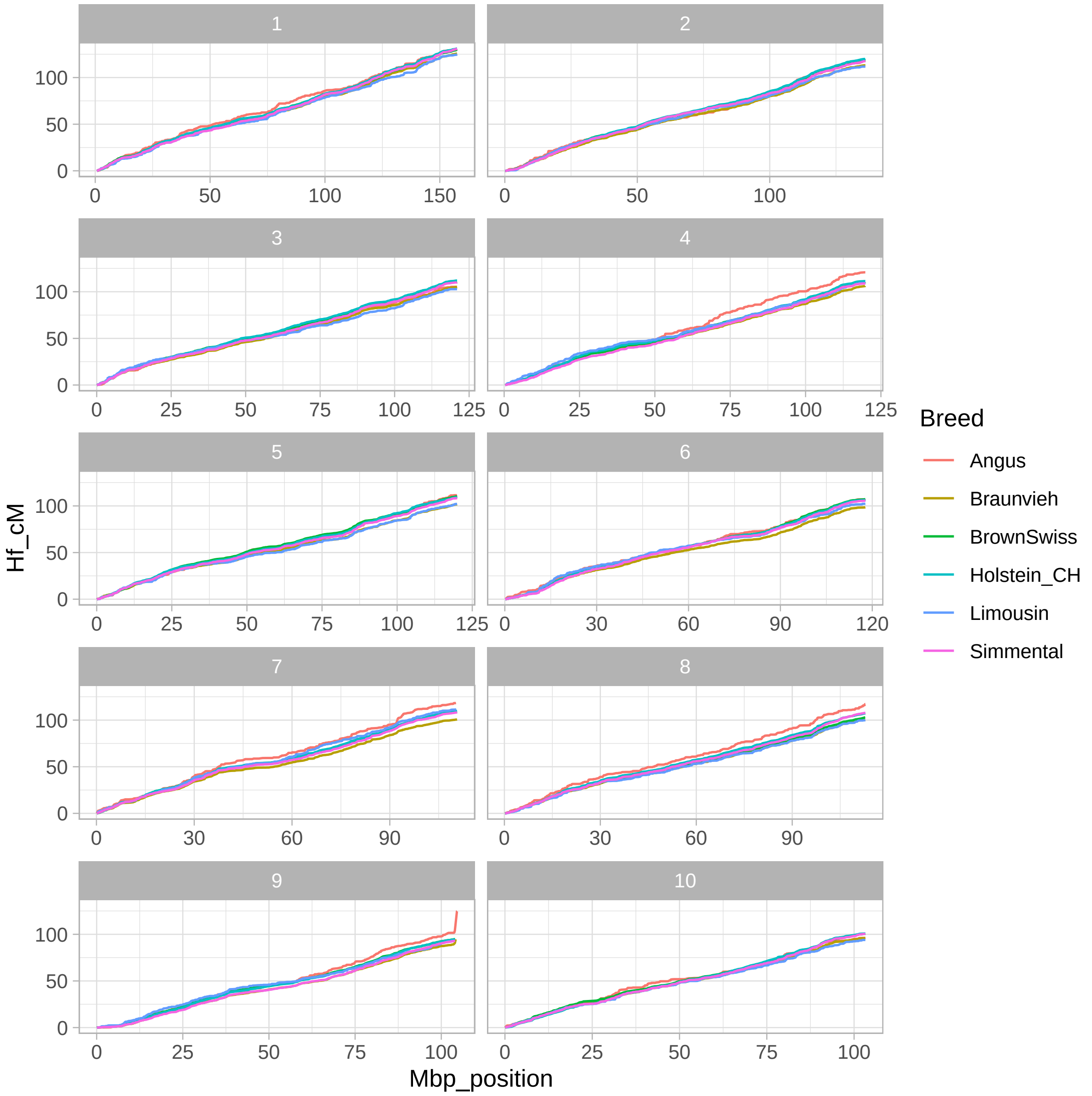

Figure S6: Female genetic map for every chromosome derived from the HMM-based approach for 6 cattle breeds.

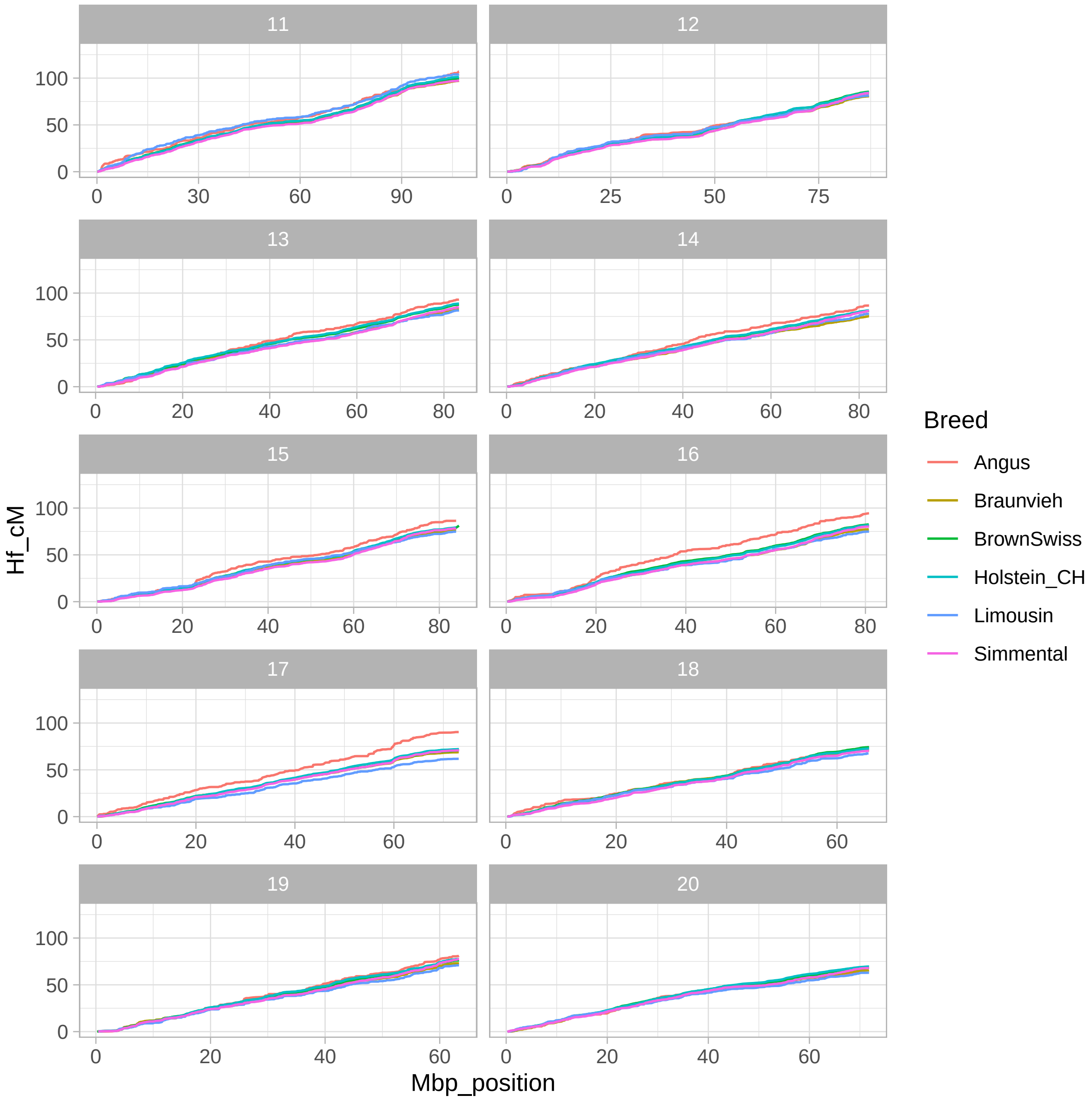

Figure S6: (cont.)

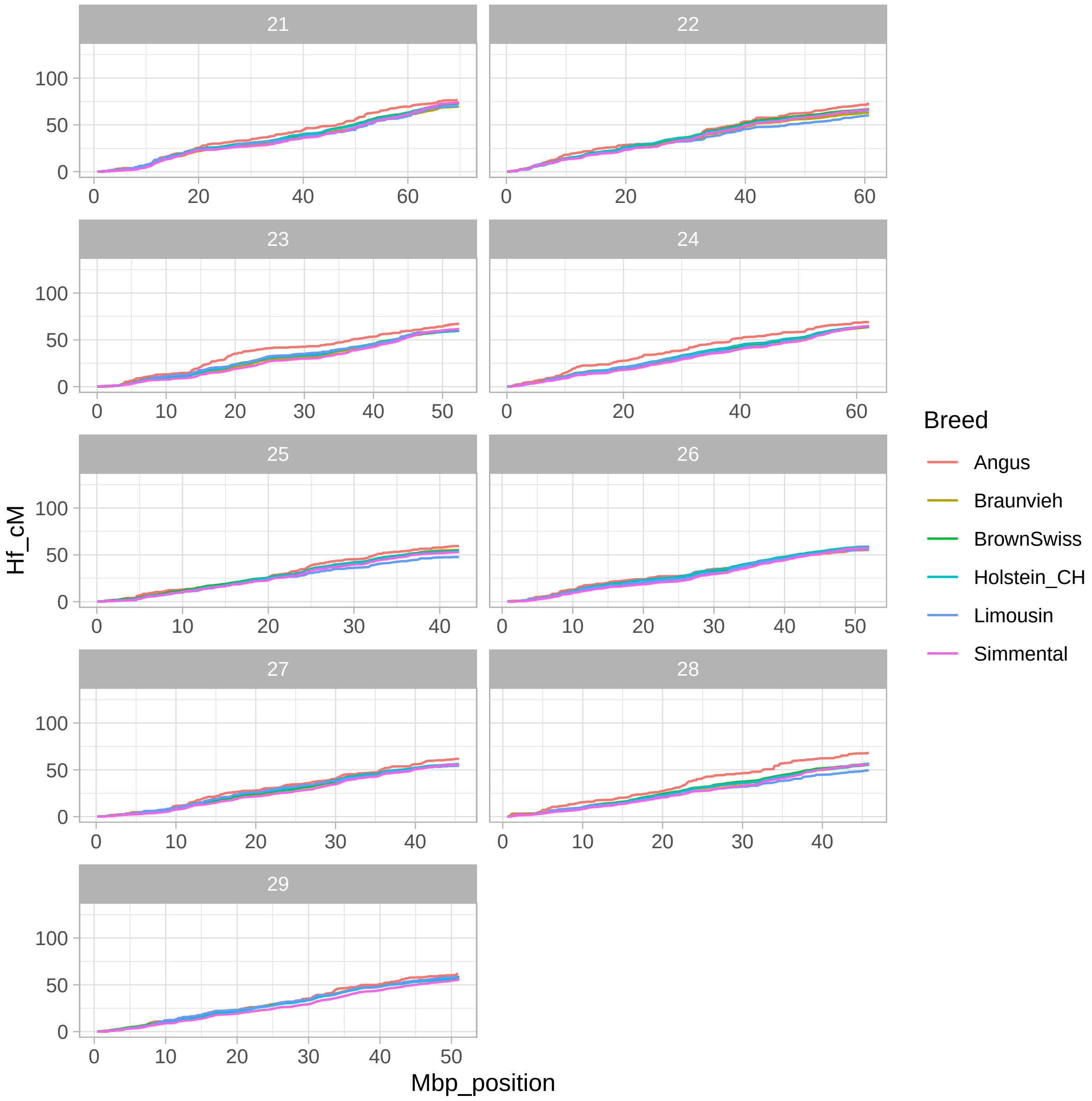

Figure S6: (cont.)

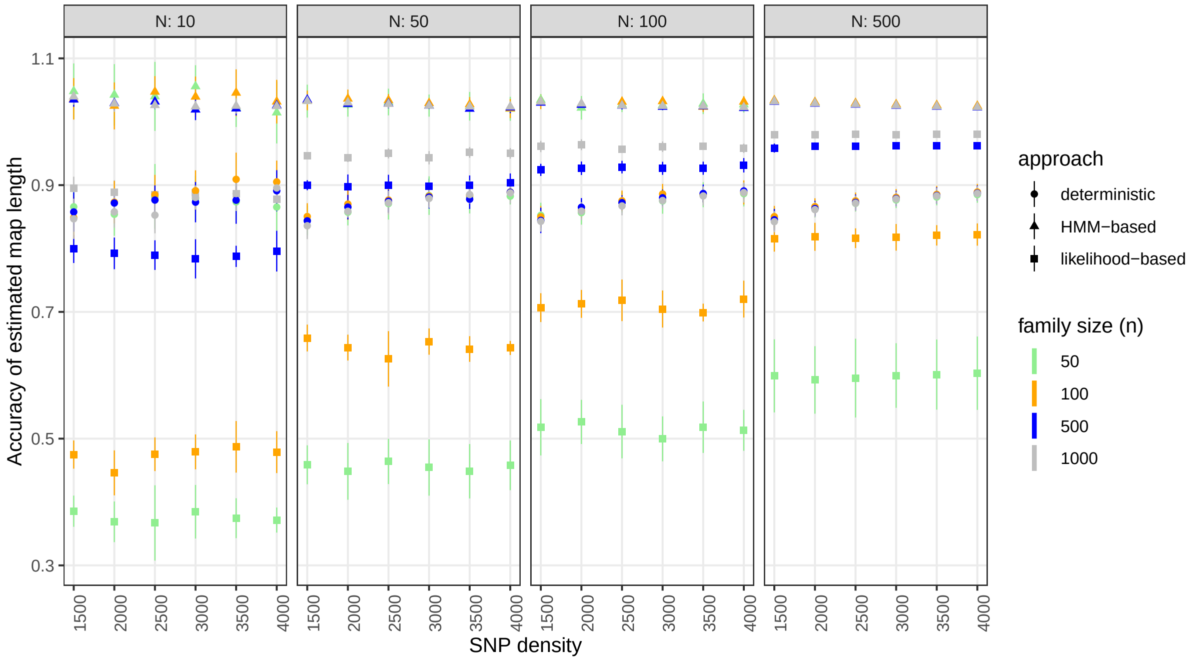

(a)

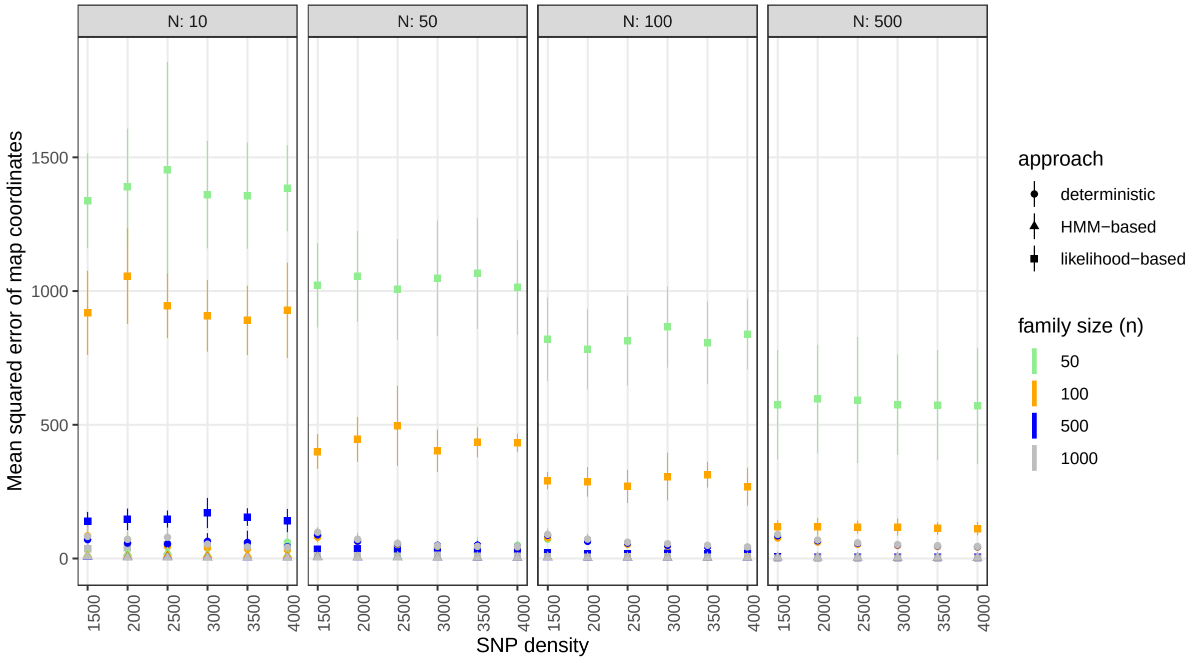

(b)

Figure S7: (a) Accuracy of map length and (b) mean squared error of genetic-map coordinates in a simulation study based on a synthetic population. Different panels refer to different numbers of half-sib families (N) used in the evaluation. The vertical coloured lines reflect the standard deviation of accuracy and mean squared error observed in 10 repetitions of simulation.

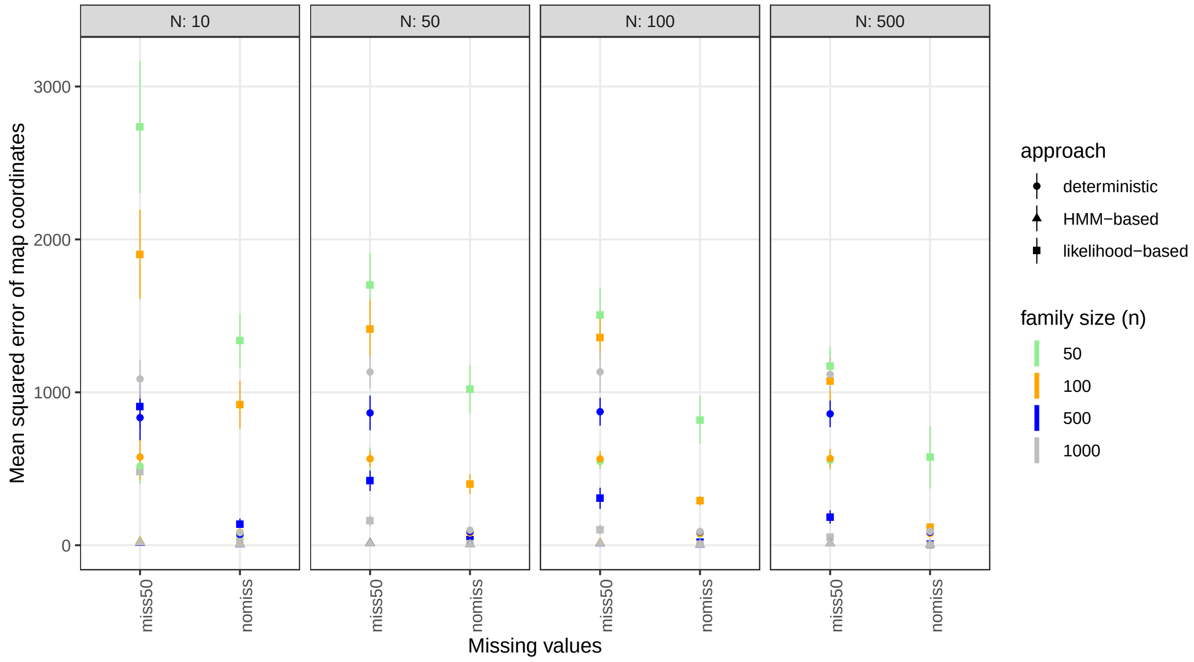

(a)

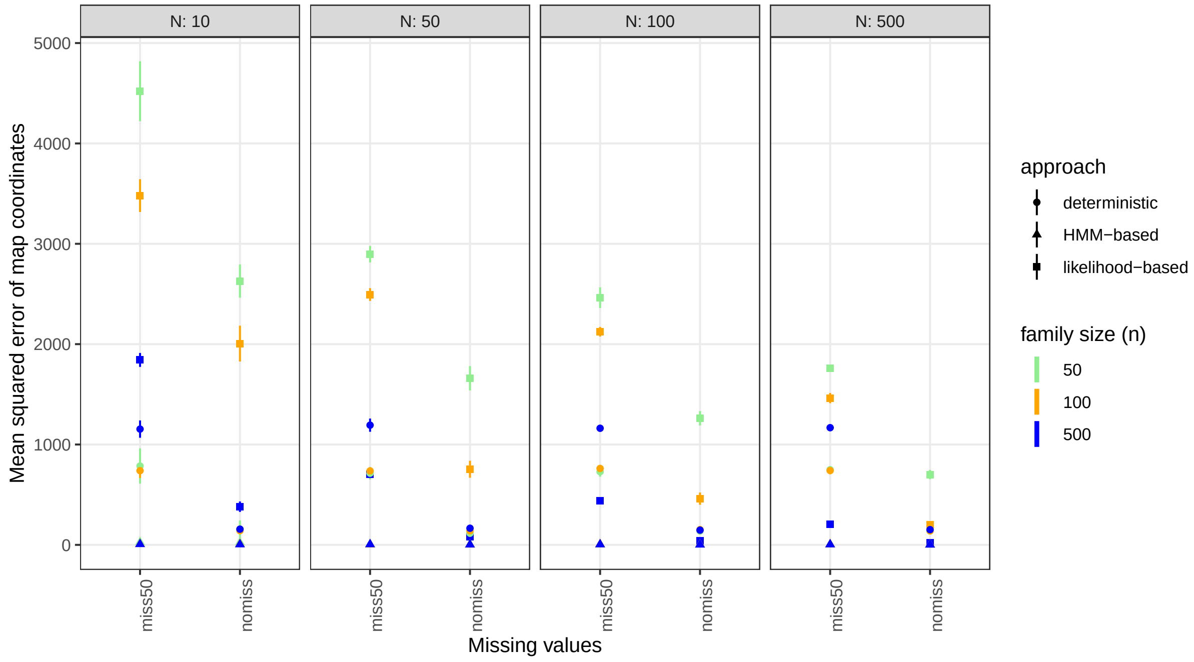

(b)

Figure S8: Mean squared error of genetic-map coordinates in a simulation study based on (a) a synthetic population (1,500 SNPs) and (b) a semi-real population (2,403 SNPs) without ("nomiss") or with 50 % ("miss50") missing genotypes. Different panels refer to different numbers of half-sib families (N) used in the evaluation. The vertical coloured lines reflect the standard deviation of mean squared error observed in 10 repetitions of simulation.

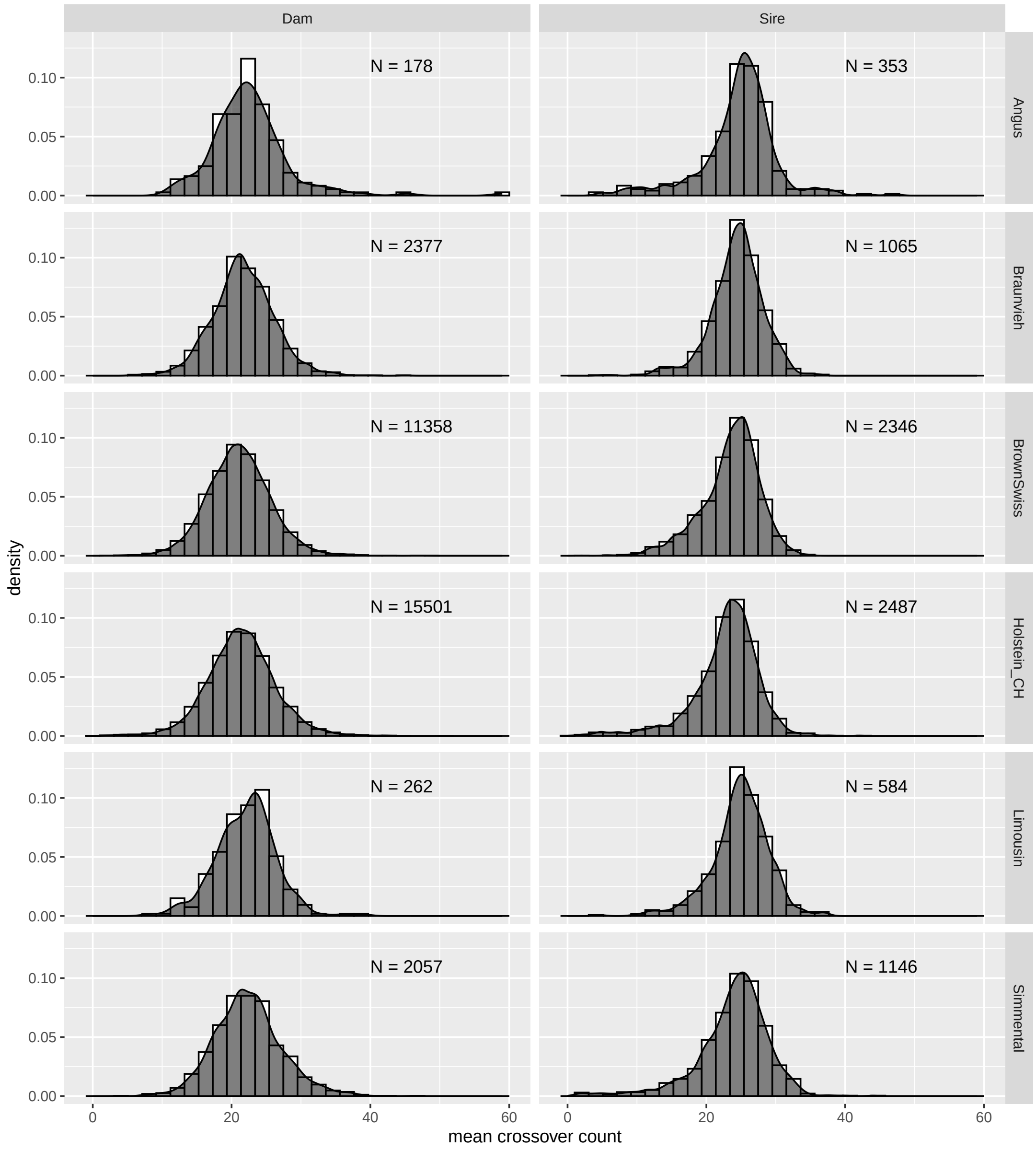

Figure S9: Histogram of mean autosomal crossover count per parent; N denotes the number of sires (right panel) and dams (left panel) that could actually be considered by the HMM-based approach.

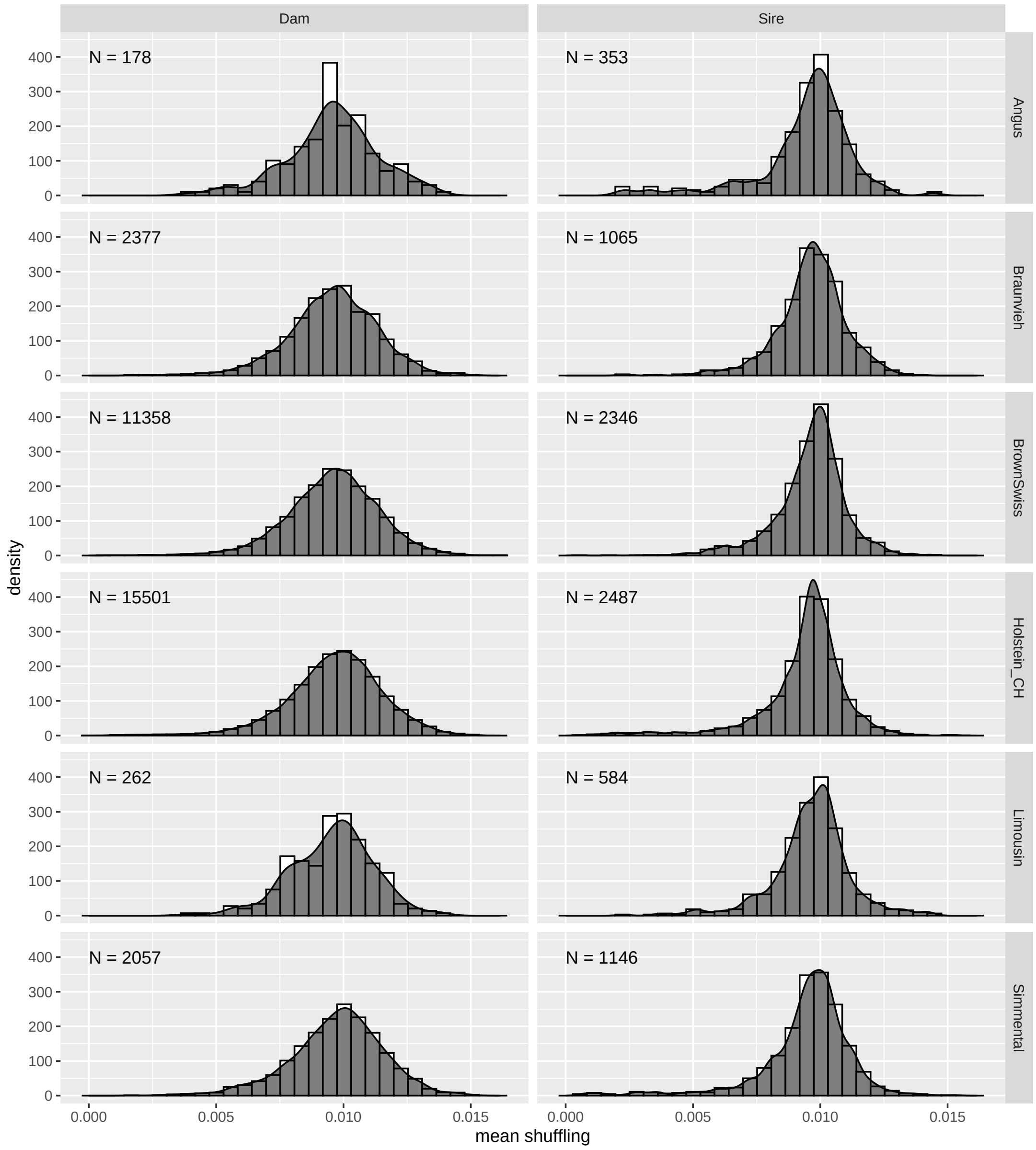

Figure S10: Histogram of mean intra-chromosomal allelic shuffling per parent; N denotes the number of sires (right panel) and dams (left panel) that could actually be considered by the HMM-based approach.

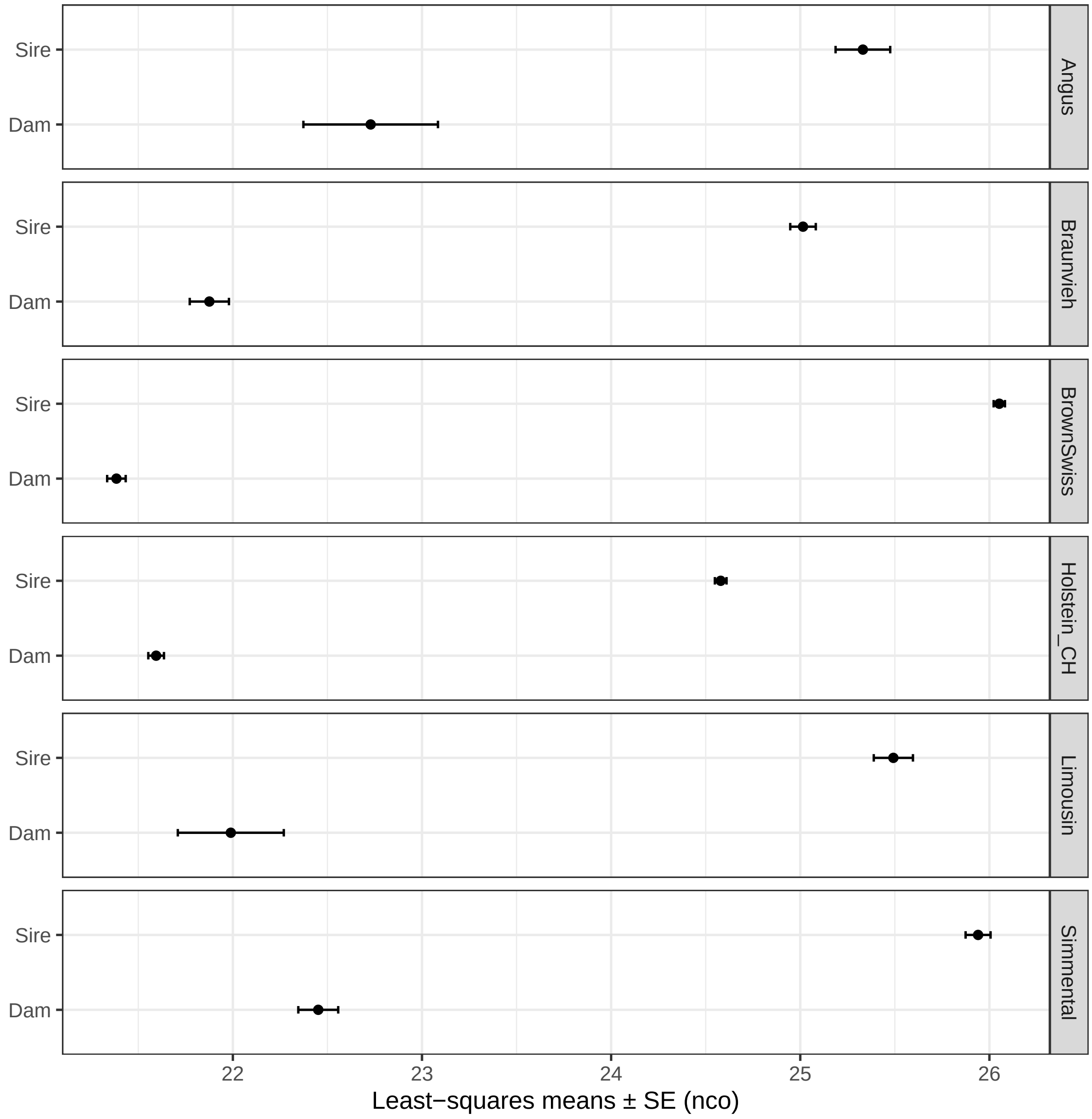

Figure S11: Least-squares means of mean autosomal crossover count per parent and breed.

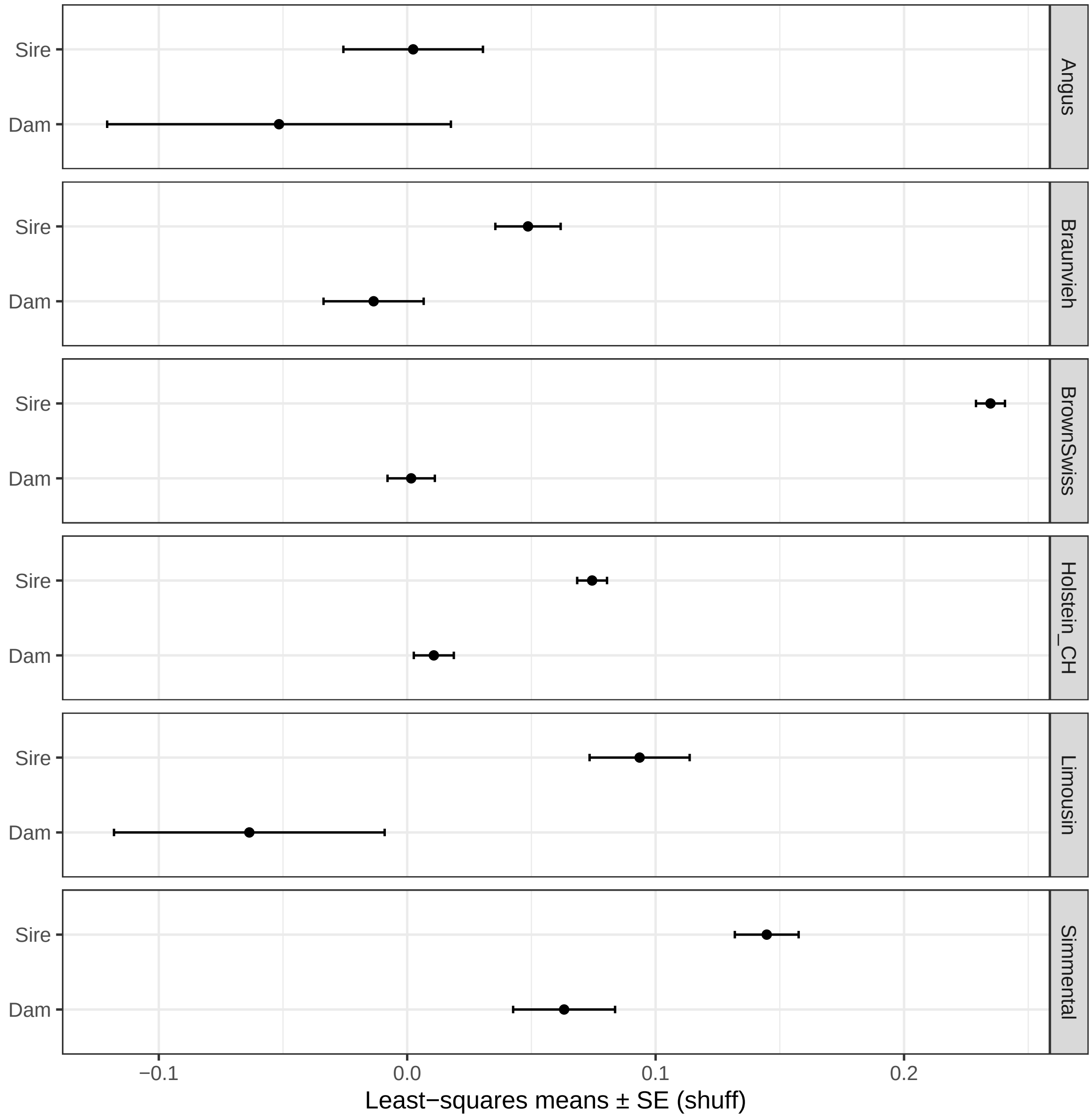

Figure S12: Least-squares means of mean intra-chromosomal allelic shuffling per parent and breed.

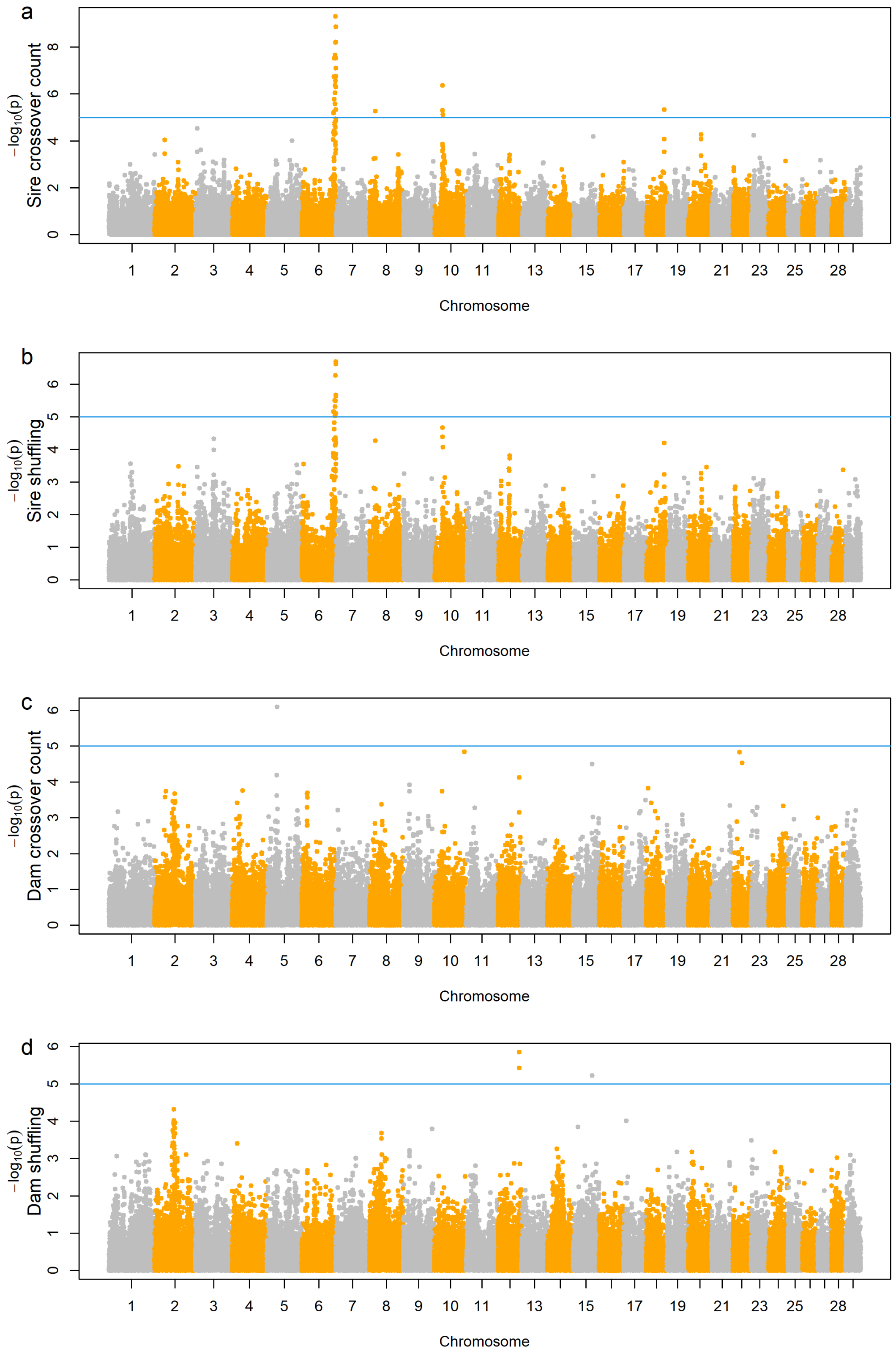

Figure S13: Manhattan plot of GWAS results in Brown Swiss; (a) autosomal crossover count of sires, (b) intra-chromosomal allelic shuffling of sires, (c) autosomal crossover count of dams, (d) intra-chromosomal allelic shuffling of dams.

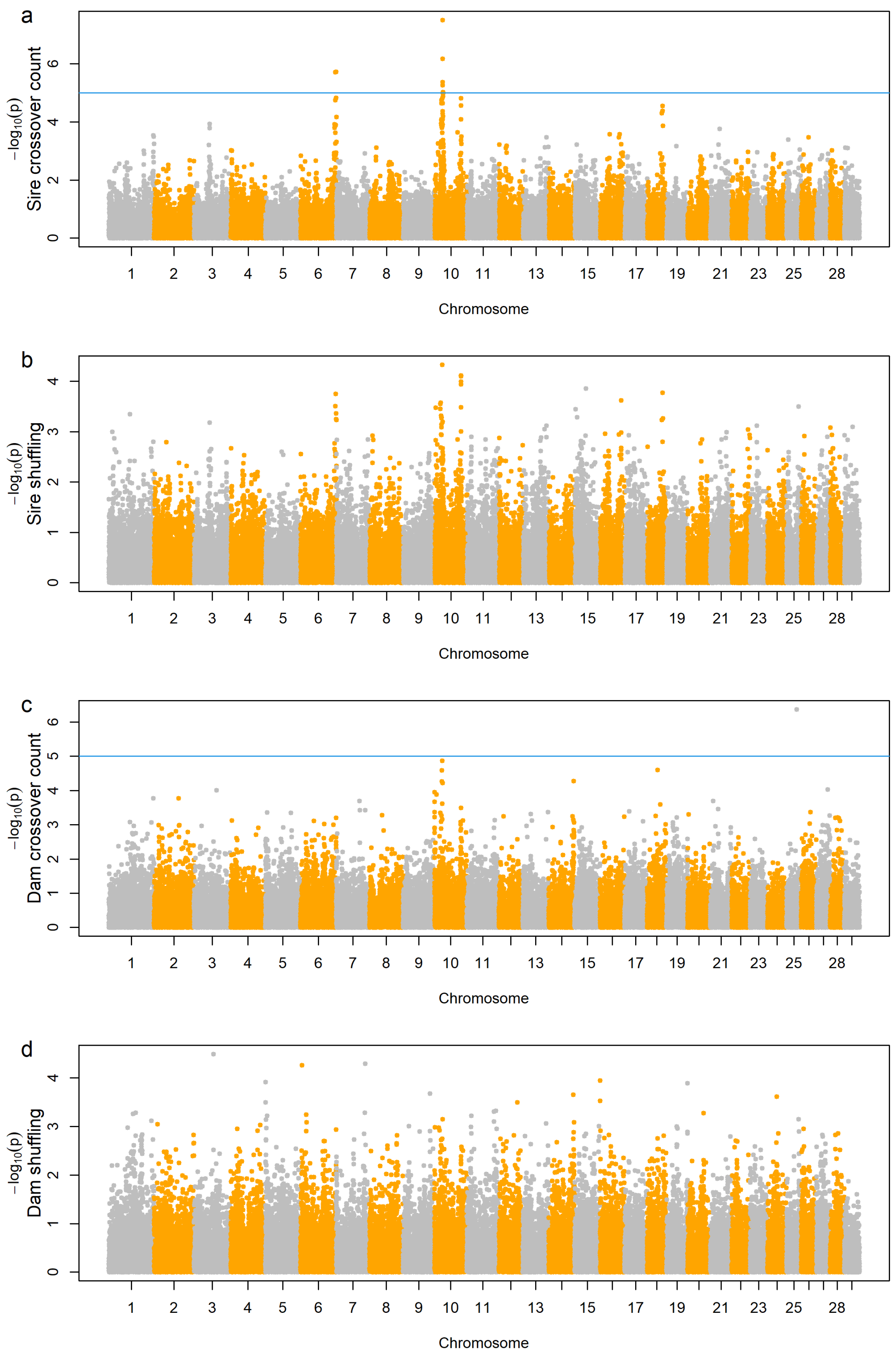

Figure S14: Manhattan plot of GWAS results in Holstein-CH; (a) autosomal crossover count of sires, (b) intra-chromosomal allelic shuffling of sires, (c) autosomal crossover count of dams, (d) intra-chromosomal allelic shuffling of dams.

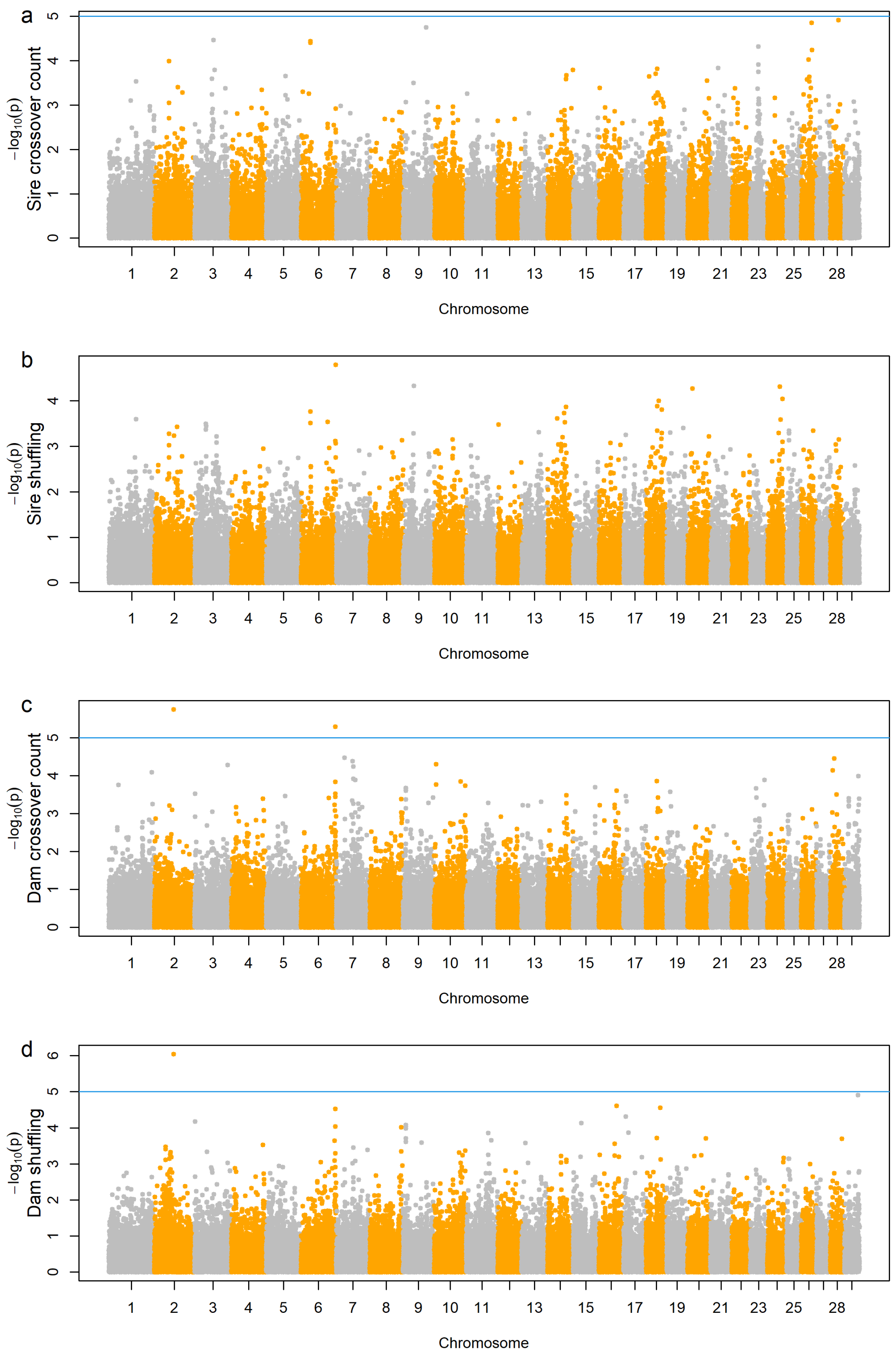

Figure S15: Manhattan plot of GWAS results in Original Braunvieh; (a) autosomal crossover count of sires, (b) intra-chromosomal allelic shuffling of sires, (c) autosomal crossover count of dams, (d) intra-chromosomal allelic shuffling of dams.

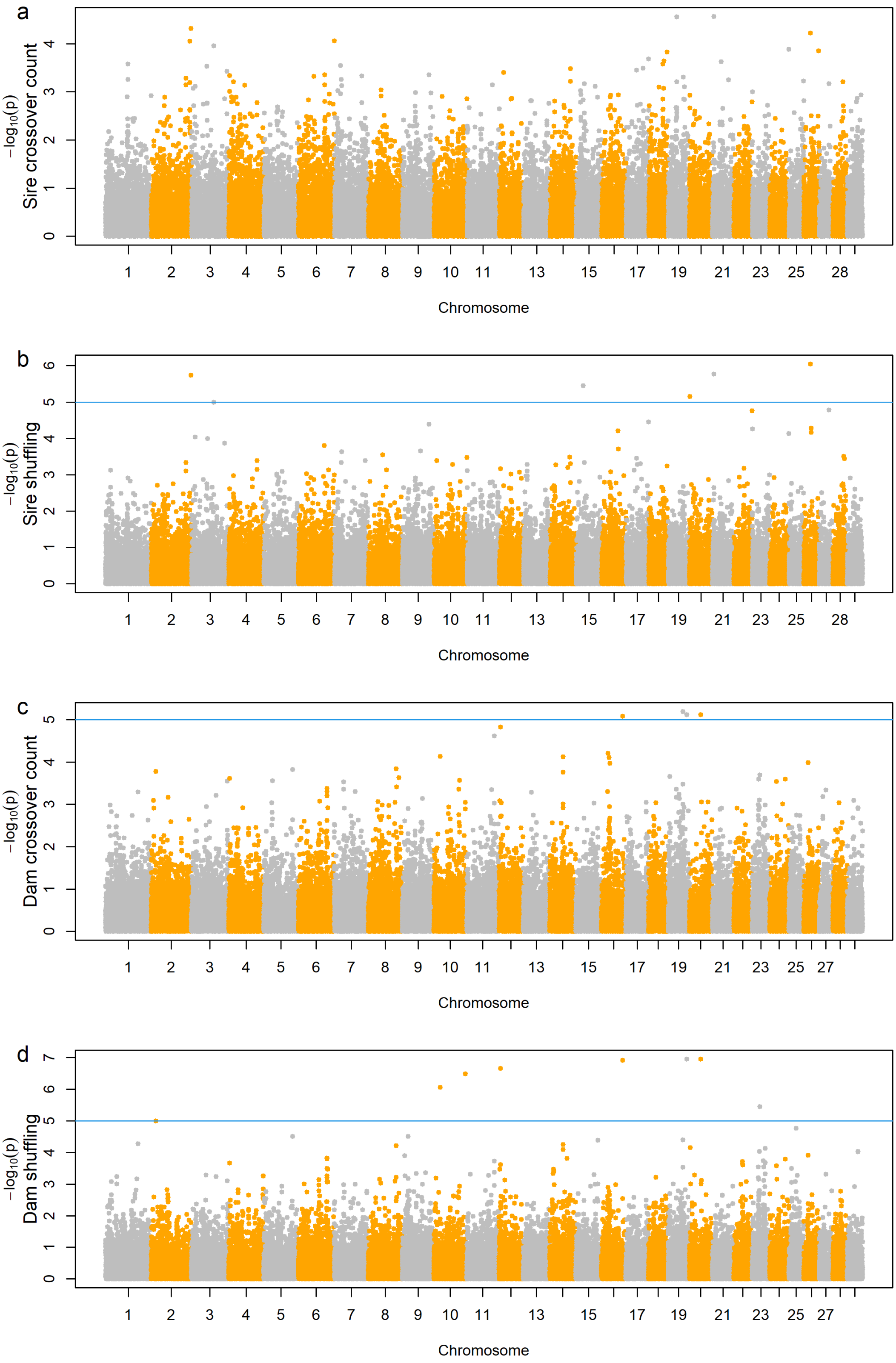

Figure S16: Manhattan plot of GWAS results in Simmental; (a) autosomal crossover count of sires, (b) intra-chromosomal allelic shuffling of sires, (c) autosomal crossover count of dams, (d) intra-chromosomal allelic shuffling of dams.

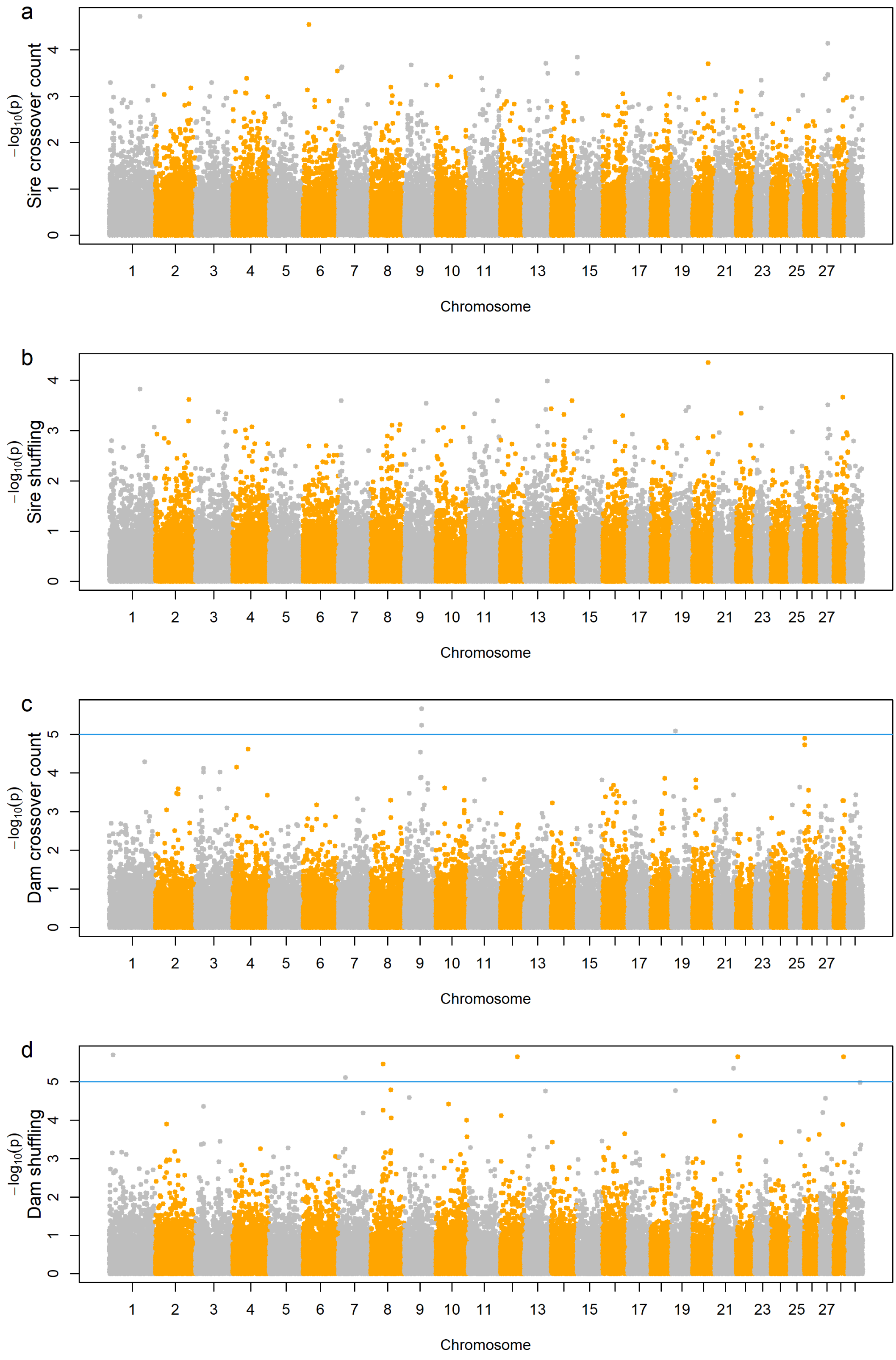

Figure S17: Manhattan plot of GWAS results in Limousin; (a) autosomal crossover count of sires, (b) intra-chromosomal allelic shuffling of sires, (c) autosomal crossover count of dams, (d) intra-chromosomal allelic shuffling of dams.

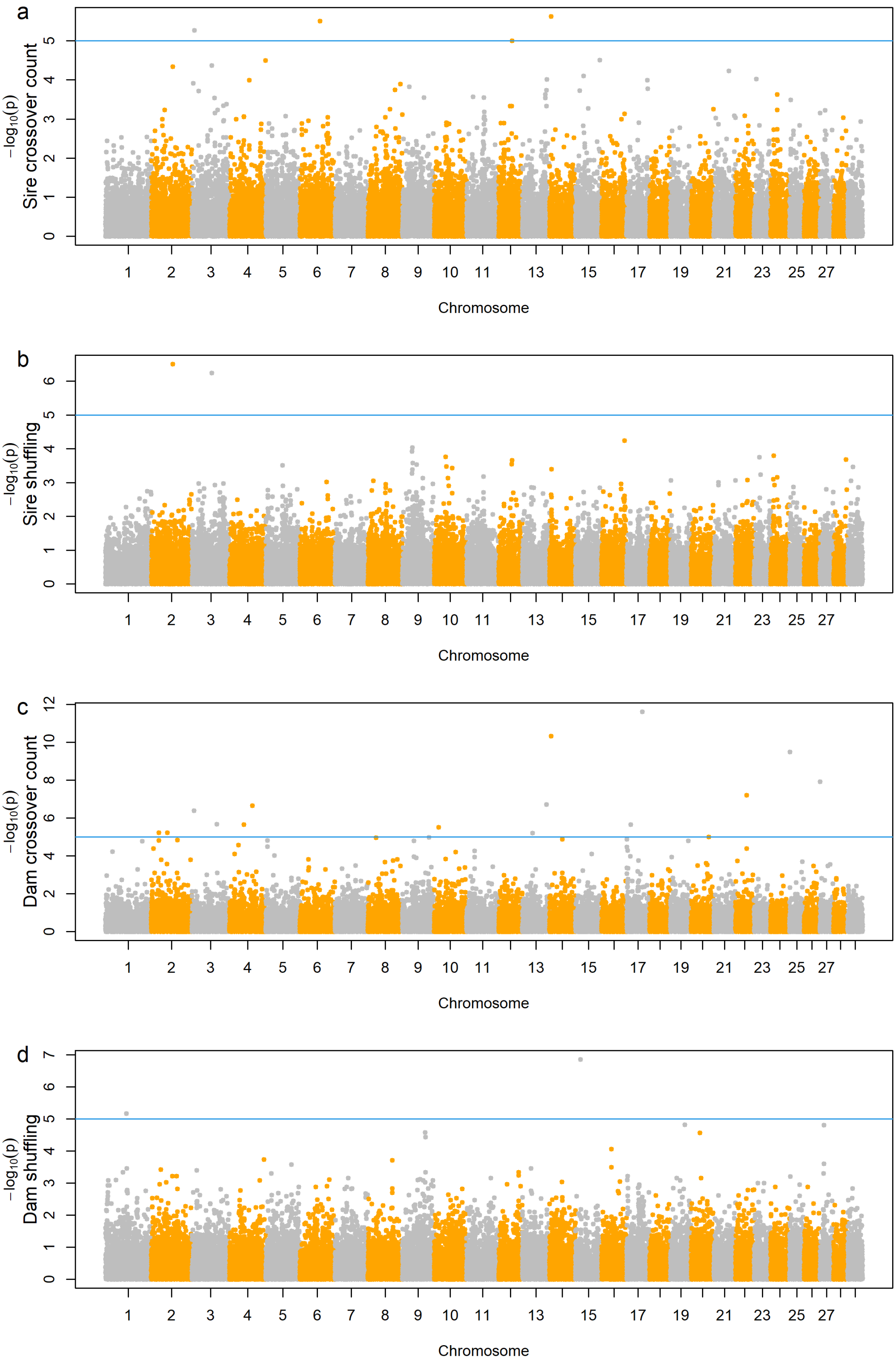

Figure S18: Manhattan plot of GWAS results in Angus; (a) autosomal crossover count of sires, (b) intra-chromosomal allelic shuffling of sires, (c) autosomal crossover count of dams, (d) intra-chromosomal allelic shuffling of dams.

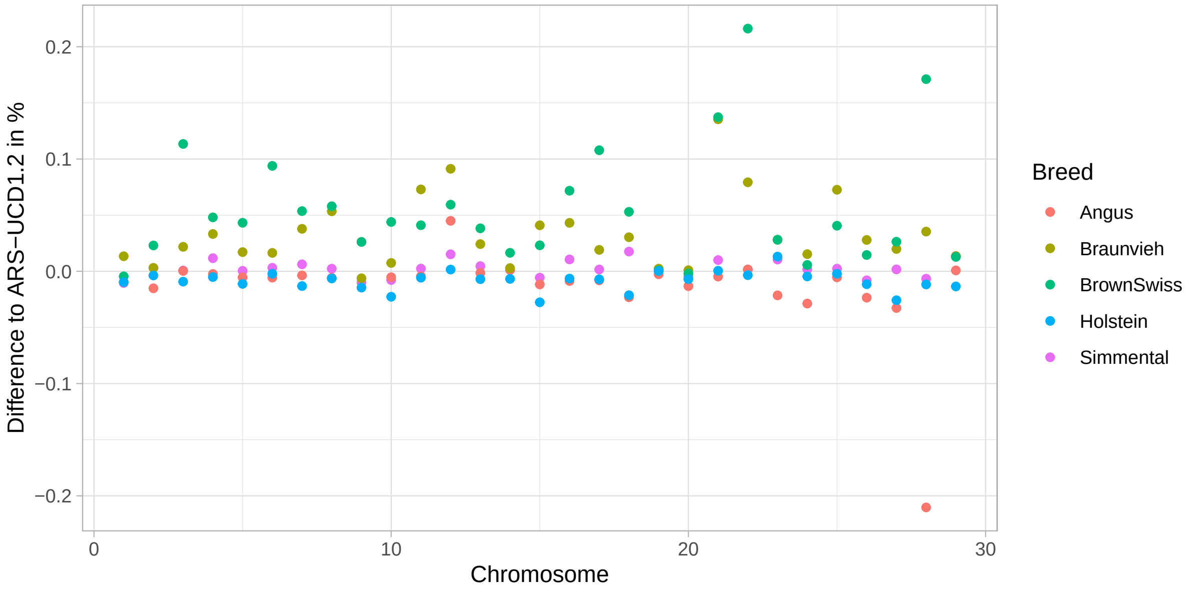

Figure S19: Deviation of physical chromosome length of breed-specific assemblies from ARS-UCD1.2 genome assembly (Hereford).

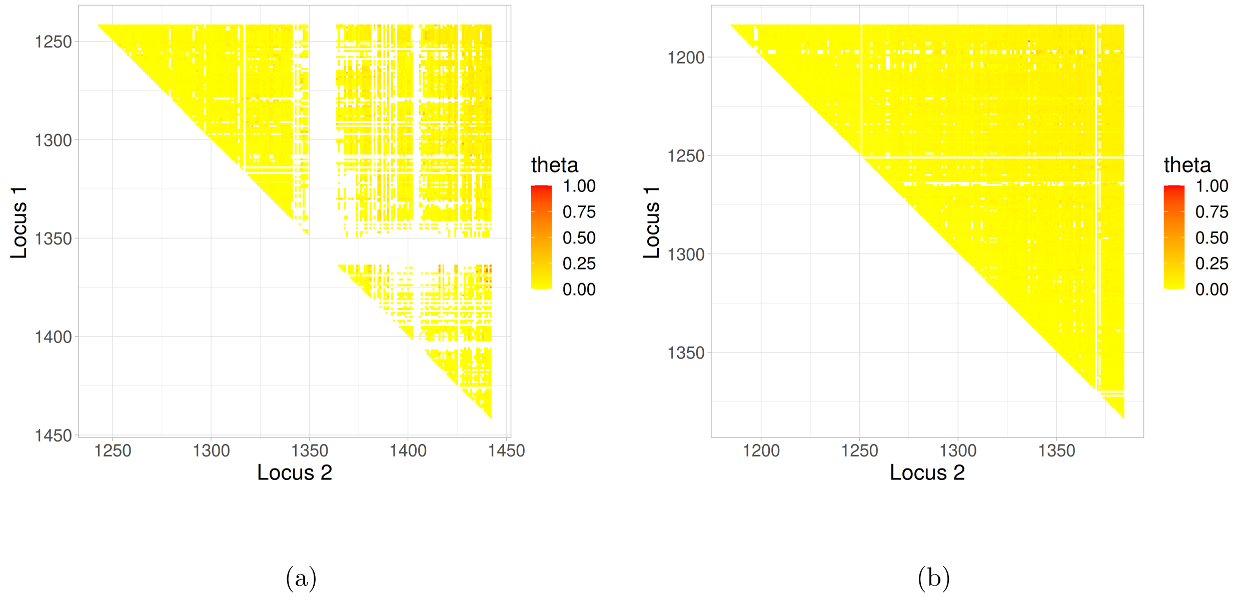

Figure S20: Recombination rate (theta) between 101 SNP pairs in the region of selection signature found in Original Braunvieh (Bhati et al., 2020). Plots are centered around 66.1 Mbp on BTA11 for (a) Original Braunvieh (60.3-72.9 Mbp) and (b) Brown Swiss (60.3-71.1 Mbp).

References

M. Bhati, N. K. Kadri, D. Crysnanto, and H. Pausch. Assessing genomic diversity and signatures of selection in original braunvieh cattle using whole-genome sequencing data. *BMC genomics*, 21(1):1–14, 2020. doi: 10.1186/s12864-020-6446-y.
